# Molecular features similarities between SARS-CoV-2, SARS, MERS and key human genes could favour the viral infections and trigger collateral effects

**DOI:** 10.1038/s41598-021-83595-1

**Published:** 2021-02-18

**Authors:** Lucas L. Maldonado, Andrea Mendoza Bertelli, Laura Kamenetzky

**Affiliations:** 1 IMPaM, CONICET, Facultad de Medicina, Universidad de Buenos Aires, Ciudad Autónoma de Buenos Aires, Argentina; 2 Universidad de Buenos Aires, Ciudad Autónoma de Buenos Aires, Argentina; 3 iB3 | Instituto de Biociencias, Biotecnología y Biología traslacional, Departamento de Fisiologia y Biologia Molecular y Celular, Facultad de Ciencias Exactas y Naturales, Universidad de Buenos Aires, Ciudad Autónoma de Buenos Aires, Argentina

**Keywords:** Viral infection, Functional clustering

## Abstract

In December 2019, rising pneumonia cases caused by a novel β-coronavirus (SARS-CoV-2) occurred in Wuhan, China, which has rapidly spread worldwide, causing thousands of deaths. The WHO declared the SARS-CoV-2 outbreak as a public health emergency of international concern, since then several scientists are dedicated to its study. It has been observed that many human viruses have codon usage biases that match highly expressed proteins in the tissues they infect and depend on the host cell machinery for the replication and co-evolution. In this work, we analysed 91 molecular features and codon usage patterns for 339 viral genes and 463 human genes that consisted of 677,873 codon positions. Hereby, we selected the highly expressed genes from human lung tissue to perform computational studies that permit to compare their molecular features with those of SARS, SARS-CoV-2 and MERS genes. The integrated analysis of all the features revealed that certain viral genes and overexpressed human genes have similar codon usage patterns. The main pattern was the A/T bias that together with other features could propitiate the viral infection, enhanced by a host dependant specialization of the translation machinery of only some of the overexpressed genes. The envelope protein E, the membrane glycoprotein M and ORF7 could be further benefited. This could be the key for a facilitated translation and viral replication conducting to different comorbidities depending on the genetic variability of population due to the host translation machinery. This is the first codon usage approach that reveals which human genes could be potentially deregulated due to the codon usage similarities between the host and the viral genes when the virus is already inside the human cells of the lung tissues. Our work leaded to the identification of additional highly expressed human genes which are not the usual suspects but might play a role in the viral infection and settle the basis for further research in the field of human genetics associated with new viral infections. To identify the genes that could be deregulated under a viral infection is important to predict the collateral effects and determine which individuals would be more susceptible based on their genetic features and comorbidities associated.

## Introduction

Since its initial outbreak at Huanan Seafood Wholesale Market in Wuhan, China, in late 2019, COVID-19 has affected more than 4 million people and caused more than 300 thousand deaths all around the world. Thereafter, scientists are focused not only on studying the biology and dissemination of COVID-19 to control the transmission and design proper diagnostic tools and treatments, but also they are racing to design a vaccine that could prevent the infection caused by the coronavirus SARS-CoV-2. This virus belongs to the *Betacoronavirus* (β-coronavirus) of the *Coronaviridae* family, which is also composed of three more genera: *Alphacoronavirus* (αCoV), *Gammacoronavirus* (γCoV), and *Deltacoronavirus* (δCoV)^1^. Viruses from this family possess a single-stranded, positive-sense RNA and the genome ranges from 26 to 32 kb^2^.

Coronaviruses have been identified in several host species including humans, bats, civets, mice, dogs, cats, cows, and camels^3–6^. Since severe acute respiratory syndrome (SARS), caused by the coronavirus SARS-CoV, emerged in southern China in 2002^7^, several studies tracing the transmission and possible reservoirs for viruses have been performed. In early 2007, it had already been warned that bats were a natural reservoir for an increasing number of emerging zoonotic viruses as well as for many viruses that have a close genetic relationship with the coronaviruses that cause the severe acute respiratory syndrome. The high genetic mutation rate of the coronaviruses increases the human and domestic mammals likelihood of getting diseases that is also worsened by legal and illegal trading of wildlife animals due to these places propitiate the environment for cross-species virus transmission contributing to the rapid spread of the viral infections around the world^6,8^. SARS and the Middle East respiratory syndrome (MERS)^9^ are genetically diverse coronaviruses that also originated from bats in wildlife trading markets with poor hygienic conditions^10^. Currently, the outbreak of an atypical pneumonia caused by the novel coronavirus SARS-CoV-2 appears to have also started from a zoonotic and a cross-species virus transmission at a market in Wuhan including bats and pangolins, where animals were kept together and the meat was sold^11^.

In order to contribute to the insight of the virus and its molecular features, here we provide a thorough and comprehensive analysis of codon usage and the molecular features of the viral genes and highly expressed genes in human lung tissue that allowed us to find particular similarities between specific viral and human genes that could help to understand the viability of the virus as well as the susceptibility of the humans to the viral infection based on the molecular features of their genes.

Codon usage bias is a phenomenon where synonymous codons are not used with equal frequency during translation of genes and it is common in a wide variety of organisms, including prokaryotes and eukaryotes^12–14^. Research on the codon usage, its causes and consequences, as well as the identification of the evolutionary forces that intervene in evolution are relevant in genomic studies to understand the biology of any organism and for the accurate application of methodologies such as heterologous gene expression^15,16^, the design of degenerate primers^17^, the prediction of gene functions^18^ and the design of attenuated vaccines^19–21^. The study of the patterns of the CUB is also useful to predict genes with high expression levels. This relies on the fact that codon usage bias of highly expressed genes need abundant ribosomes and tRNAs matching properly for an efficient translation conducing to the optimization of particular codons for the translation of particular genes^22–27^. Since viruses replicate inside of living cells and depend exclusively on the protein synthesis machinery and chaperones of their hosts, the primary structure of viral genes could be determined by the same forces that shape the codon usage in their hosts and if no other evolutionary force shapes the molecular features and codon usage preferences of the viral genes, they would be a reflection of the host machinery. However, the viral codon usage evolution is more complex and other factors such as mutation pressure, particular DNA/RNA or protein structure and genome size are also involved^28,29^. The codon usage frequency varies significantly among genes within the same organism as well as in viruses. In a multicellular host, viruses are normally restricted to specific organ, tissue, or cell type^29^. Many studies suggest that human viruses have CUB that match highly expressed proteins in the tissues they infect^30,31^. Codon pair bias and dinucleotide preferences of viruses have been suggested as the main factors that reflect the codon usage of their hosts. Indeed, virus attenuation by codon pair deoptimization is used as an efficacious attenuation method of various small RNA viruses and has resulted in the generation of superior experimental live virus vaccines^20,32–36^.

Principal component analysis (PCA) and hierarchical clustering are unsupervised machine learning methods widely applied in many codon usages studies that allow to resolve the high-dimensional molecular and codon usage features by reducing them to a limited number of variables and build associations based on the parametric features. Hereby, our work revealed molecular and evolutionary aspects of the human coronaviruses SARS-CoV-2, SARS and MERS that helps to determine whether the level of similarity of the codon usage and the molecular features between the highly expressed genes in human lung tissue and the genes of the coronaviruses are responsible for the codons selection in the viruses and whether these could propitiate viral infections. The correlation between some of the overexpressed human genes and some viral genes suggested that the translation machinery of the host contributes partially to the fitness of the viruses. Furthermore, those genes whose molecular features are more similar to the viral genes could contribute more to propitiate the system for the viral replication and for modelling the viral gene features. Since the molecules of the translation machinery of the overexpressed genes should be in the proper abundance to fit the needs of the protein translation of the host, the virus can take advantage from this to synthetise their own particles to the detriment of the normal function of the host cells.

## Methods

Up to late April, a total of ⁓ 500 SARS-CoV-2 β-coronavirus genomes became available. The total available sequences of β-coronavirus were downloaded from the NCBI (https://www.ncbi.nlm.nih.gov/labs/virus/vssi/#/) including the reference genomes of MERS (NC_019843), SARS (NC_004718) and SARS-CoV-2 (NC_045512) and were classified according to their host. Different SARS-CoV-2 isolates from different countries were pre-analysed, but only reference genomes were retained due to the low variability of the data. The genomes quality was assessed and the genomes containing more than 10 gaps were discarded. CDS of representative viruses from the previous classification were selected and analysed. For the analysis of human genes, we selected 463 highly expressed human genes in lung tissue according to the fold change between the expression level in lung and the tissue with the second highest expression level according to the “Human Protein Atlas” (https://www.proteinatlas.org/humanproteome/tissue/lung). To probe that the clustering and the differences observed were not produced by chance, the lung under-expressed human genes were used as a control to compare their codon features against the viral genes of the human SARS, SARS-CoV-2 and MERS. Furthermore, the viral genes of bats and pangolins SARS-CoV-2-like viruses, bats and civets SARS-like viruses and bats and hedgehogs MERS-like viruses were also used as control to compare the codon features against those of the overexpressed human genes. We considered valid CDS when they started with an ATG codon, ended with an in-frame stop codon, and had no undetermined nucleotides nor internal stop codons. The accession numbers of the sequences that were used here can be found in Supplementary Information 1.

The CUB analyses were performed with CodonW 1.4.4 (J Peden, http://codonw.sourceforge.net/). The total GC content of the CDS as well as the GC content of the first (P1), second (P2), and third (P3) codon positions were calculated using custom PERL scripts. To correct the inequality composition at the third codon position^37^, the three stop codons (UAA, UAG, and UGA) were excluded in the calculation of P3, and the two single codons for methionine (AUG) and tryptophan (UGG) were excluded from P1, P2, and P3.

### Codon usage indices

The following codon indices were calculated: relative synonymous codon usage (RSCU)^38^, the effective number of codons (ENc)^39^, codon adaptation index (CAI)^38,40^, codon bias index (CBI)^27^, the optimal frequency of codons (Fop)^41^, General Average Hydropathicity (GRAVY)^38^, aromaticity (Aromo)^42^ and GC-content at the first, second and third codon positions (GC1, GC2 and GC3), frequency of either a G or C at the third codon position of synonymous codons (GC3s), the average of GC1 and GC2 (GC12) and Translational selection (TrS2).

ENc indicates the degree of codon bias for individual genes. Over a range of values from 20 to 61, lower values indicate higher codon bias, while ENc equal to 61 means that all codons are used with equal probability^39,43^.

CAI values measure the extent of bias toward preferred codons in highly expressed genes. CAI values range between 0 and 1.0, with higher CAI values indicating higher expression and higher CUB^38,40^ under the assumption that translational selection would optimize gene sequences according to their expression levels.

CBI is another measure of directional codon bias, based on the degree of preferred codons used in a gene, like the frequency of optimal codons. It measures the extent to which a gene uses a subset of optimal codons. In genes with extreme codon bias, CBI will be equal to 1, whereas in genes with random codon usage the CBI values will be equal to 0^27^.

Fop is a species-specific measure of bias towards particular codons that appear to be translationally optimal in particular species. It can be calculated as the ratio between the frequency of optimal codons and the total number of synonymous codons. Its values range from 0 if a gene contains no optimal codons to 1 if a gene is entirely composed of optimal codons^41^. The determination of optimal codons was carried out based on the axis 1 ordination, the top and bottom 5% of genes were regarded as the high and low bias datasets, respectively. Codon usage in the two data sets was compared using chi-square tests, with the sequential Bonferroni correction to assess significance according to Peden^44^. Optimal codons were defined as those that are used at significantly higher frequencies (p-value < 0.01) in highly expressed genes compared with the frequencies in genes expressed at low levels.

GRAVY values were calculated as a sum of the hydropathy values of all the amino acids encoded by the codons in the gene product divided by the total number of residues in the sequence of the protein. The more negative the GRAVY value, the more hydrophilic the protein is, whereas while the more positive the GRAVY value, the more hydrophobic the protein^38^.

Aromo values denote the frequency of aromatic amino acids (Phe, Tyr, Trp) encoded by the codons in the gene product^42^.

TrS2 estimates the codon-anticodon interaction efficiency revealing bias in favour of optimal codon-anticodon energy and represents the translational efficiency of a gene. TrS2 value > 0.5 shows bias in favour of translational selection according to Gouy and Gautier^45–47^.

### Codon pair score and codon pair bias

The determination of the CPB in the coding sequences was performed using CPBias (https://rdrr.io/github/alex-sbu/CPBias/) developed in R. as described by Coleman et al.^20^. The CPS is defined as the natural logarithm of the ratio of the observed over the expected number of occurrences of a particular codon pair in all protein-coding sequences of a species. The CPB was used as an index but also to determine the bias in CPS between the virus and the hosts genes. The expected number of codon pair occurrences estimates the number of codon pairs to be present if there is no association between the codons that form that codon pair. It is also calculated to be independent of codon bias and amino acid frequency^20^. A negative CPS value means that a particular codon pair is underrepresented, whereas a positive CPS value indicates that a particular codon pair is overrepresented in the analysed protein-coding sequences. Codon pairs that are equally under- or overrepresented have a CPS equidistant from 0. We calculated CPS for each of the 3,721 possible codon pairs (61 × 61 codons).

### ENc-plots

The ENc-plot was used to analyse the influence of the base composition on the codon usage^48^. The ENc values were plotted against GC3s values and a standard curve was generated to show the functional relationship between ENc and GC3s values under mutational bias rather than selection pressure. In genes where codon choice is constrained only by a G + C mutational bias, the predicted ENc values will lie on or close to the GC3s standard curve. However, the presence of other factors, such as selection effects, causes the values to deviate considerably from the expected GC3s curve. The values of ENc range from 20 (when only one codon is used per amino acid) to 61 (when all codons are used with equal probability). The predicted values of ENc were calculated according to Hartl et al*.*^48^.

### Clustering analysis

A total of 91 codon usage features were extracted and used as variables for the clustering analysis of the viral genes coming from animal hosts and human host and overexpressed human genes in lung tissue, including the gene composition, RSCU frequencies and the indices described in M&M section. The variables were integrated into an input matrix to feed the clustering algorithm. These can be found in the Supplementary Information 1. Hierarchical clustering using Euclidean distances was performed. The clValid (https://www.rdocumentation.org/packages/clValid/versions/0.6-9) package clustering algorithm was used to choose and validate the best clustering method. To gain accuracy and confidence in the correlation methods and to evaluate the cluster stability, we used an iterative bootstrapping method using Flexible Procedures for Clustering (fpc: https://cran.r-project.org/web/packages/fpc/index.html), which means that after a hundred repetitions, the cluster was consistent and statistically stable. Furthermore, as a control of the clustering, we used the viral genes of SARS-CoV-2-like, SARS-like and MERS-like isolated from the animal hosts (bats, civets, hedgehogs and pangolins) and the 70 overexpressed human genes that had grouped with the viral genes of the human SARS-CoV-2, SARS, and MERS.

Also, random under expressed human genes in lung tissues according to Protein Atlas (https://www.proteinatlas.org/humanproteome/tissue/lung) and the viral genes of the human SARS, SARS-CoV-2 and MERS were used to perform clustering analysis as other control as explained before. Clustering method created genes groups based on the codon usage similarities between the viral genes and human genes. In order to evaluate the significance of the different groups’ conformation and the number of the human genes associated to the different conditions (overexpressed and under expressed genes in lung tissues) that clustered with the viral genes of both, human and animal viruses, we statistically tested it using the chi-square test of independence.

### Principal component analysis (PCA)

PCA was used to evaluate the codon usage variation among the genes as the multivariate statistical method. The axes represent and allow to identify the most prominent factors contributing to the variation among the genes. Since there are a total of 59 synonymous codons (including 61 sense codons, minus the unique Met and Trp and stop codons), the degrees of freedom were reduced to 40 at removing variations caused by the unequal usage of amino acids during the correspondence analysis of RSCU^49^. The data were normalized according to Sharp and Li^38^ in order to define the relative adaptiveness of each codon^44,50^, codon usage indices described above were also included as variables. PCA analyses were performed using “factoextra R package” (https://cloud.r-project.org/web/packages/factoextra/index.html).

### Phylogenetic analyses

The DNA genome sequences of all the viruses were aligned using ClustalO v1.2.4^51^. PartitionFinder 2^52^ was used to select the best-fit partitioning schemes and models of evolution for the phylogenetic analysis. The evolutionary model was set on generalised time-reversible substitution model with gamma-distributed rate variation across the sites and a proportion of invariable sites (GTR + G). The final phylogeny was calculated using fasttree^53^. The bootstrap consensus trees inferred from 1000 replicates were retained in the bootstrap and the final trees were drawn using Figtree (https://github.com/rambaut/figtree/releases).

## Results

### Phylogeny

Up to late April, a total of ⁓ 500 SARS-CoV-2 β-coronavirus genomes became available and the number of available genomes incremented substantially. The sequences of β-coronavirus were downloaded from the NCBI. In order to evaluate the variability and to select the best candidates for codon usage and nucleotides content analysis, we performed phylogenetic analyses by implementing the GTR + G model according to partition finder results. Firstly, a phylogenetic tree was constructed (data not shown) using the whole genome sequences of SARS-CoV-2 reported in humans from Spain, USA, Italy, South America, China, Korea, Japan, Australia, the *refseq* genomes of MERS and SARS and the viruses genomes that were isolated from bats, pangolins, civets, hedgehogs, *Bos taurus*, and canids (Supplementary Information 1). Since all the SARS-CoV-2 genome sequences remained together in the same cluster, we selected representative virus genomes randomly from each country and from each host and constructed the final phylogenetic tree (Supplementary Information 2). The topology of this tree showed that SARS-CoV-2 samples diverge from a common node close to the bats virus (Accession MN996532) and that all of them diverge from a common node very close to the pangolin virus (Accessions MT040333, MT040334, MT040335, MT040336, MT072864). From a distant node that contained the node of SARS-CoV-2, and that also clustered a set of bat viruses, SARS virus diverges and grouped within a node together with Civets viruses, but also adjacent and very close to other bats viruses (Accession KY417146, KT444582 and KY417150). MERS viruses grouped in a different node also very close to other cluster containing bat viruses (Accession MF593268 and KC869678). Furthermore, adjacent to this node we found hedgehogs viruses (Accession KC545383, KC545386, MK679660, MK907286, MK907287 and NC_039207).

For further analyses of molecular features and codon usage patterns of viral genes, we selected the viruses according to the phylogenetic tree described above. First, we identified the 3 nodes containing the human viruses: SARS-CoV-2, SARS and MERS. Then we selected the closest viruses to each human virus within the nodes and classified them according to their hosts (bats, civets, hedgehogs, and pangolins). From the human viruses, only the references MERS (NC_019843), SARS (NC_004718), and SARS-CoV-2 (NC_045512) were used. The total selected CDSs comprised 104 viral CDS.

### Viral gene codon usage patterns

**Figure 1:**
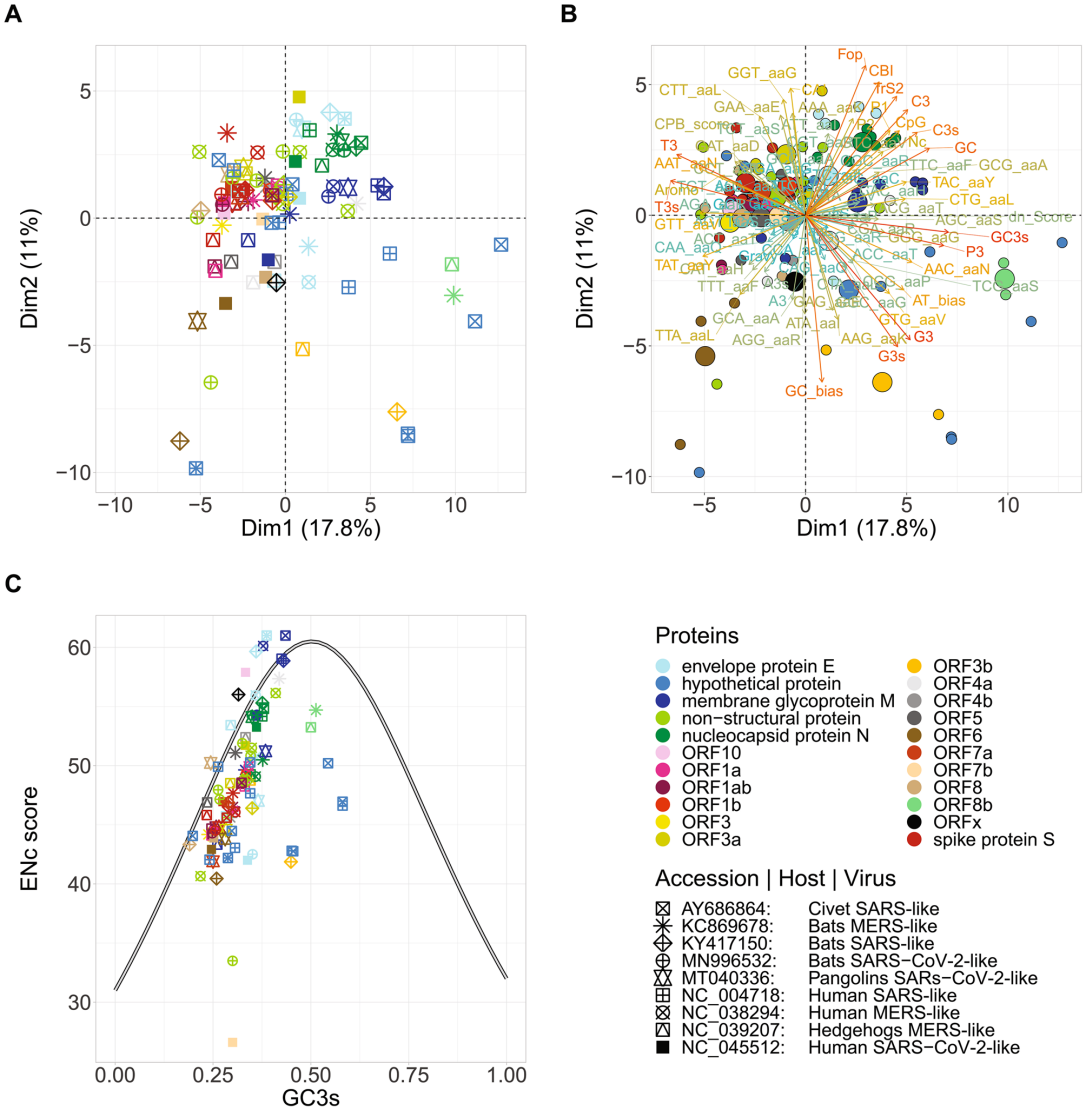
Viral genes distribution in PCA plot in the first 2 axes and ENc-GC3s plot of SARS-CoV-2 (NC_045512), SARS (NC_004718) and MERS (NC_038294), and the related virus of non-human hosts: Civets SARS-like (AY686864), bats MERS-like (KC869678), bats SARS-like (KY417150), bats SARS-CoV-2-like (MN996532), pangolins SARS-CoV-2-like (MT040336) and hedgehogs MERS-like (NC_039207). (**A**) Distribution of viral genes in PC1 and PC2. (**B**) Main factors represented by vectors that contribute to the distribution of viral genes in PC1 and PC2. (**C**) Distribution of the effective number of codons (ENc) in relation to the GC3s of viral genes. The standard curve of ENc is indicated in solid line.

PCA of codon usage and molecular features of viral genes of SARS, MERS, SARS-CoV-2, and related viral genes coming from animal hosts (bats, civets, hedgehogs, and pangolins) were performed to characterise the genes and distinguish important gene features among the viral gene families and the species. PCA allows to determine the main factors that contribute to the genes distribution providing insight of how different the genes are. Thus, the principal component with highest variance would be the best feature that would allow us to separate the data. PCA showed that the genes dispersed differentially according to the kind of gene rather than to the host that the viruses infect. Only some genes seemed to be dependent on the host (Fig. 1A,B). Most of the genes belonging to the same gene family overlapped or positioned very closely. The position of the nucleocapsid protein N is the same for all viruses except for SARS-CoV-2, which is the most distant from the group, followed by the ones of SARS and MERS. Regarding the envelope protein E, the gene of civets SARS-like and human SARS occupied the same position, while the gene of human SARS-CoV-2 and MERS distributed distantly from the group and from each other. The genes that encode for the membrane glycoprotein M also showed a distribution along positive axis 1. However, for the human SARS-CoV-2 and the hedgehogs and bats MERS-like, these genes distributed away from the genes of pangolins and bats SARS-CoV-2-like, human MERS and SARS and bats and civets SARS-like toward the inferior left quadrant. In a PCA plot the longer the vector is, the higher the contribution of the variable to the dispersion and therefore, to the differences among the genes. As indicated by the vector length, the main factors that contributed to the dispersion of these 3 genes (nucleocapsid protein N, envelope protein E, and membrane glycoprotein M) were CBI, Fop, TrS2, C3, C3s, CpG and GC. In addition, the membrane glycoprotein M seems to be more influenced by the codon frequency of the codon TAC for Tyrosine and CTG for Lysine. Whereas, for hedgehogs, MERS and the humans SARS-CoV-2, the codons TAT for Tyrosine and TTA for Lysine as well as the GC bias contributed mostly. All of them are strongly influenced by the A/T composition in the third codon position, being a common pattern for all these genes. On the other hand, the spike proteins S of all viruses distributed toward positive values of the PC2 and were also highly influenced by the A/T composition in the third codon position. All these genes grouped very closely, except the gene from bats MERS-like and human MERS. Several ORF proteins occupied the same area overlapping or distributed very close to each other. Other ORF genes such as ORF6, ORF8, and ORF3 of the human virus SARS-CoV-2 distributed distantly.

The CUB was estimated based on the ENc values. The ENc values range from 20 (when only one codon is used per amino acid) to 61 (when all codons are used with equal probability). Those genes whose ENc values are lower than 50 are considered to have skewed codon usage. All the viruses presented a wide range of ENc (26 < ENc < 58) that varied mainly depending on the type of genes or gene family. The highest ENc median was observed for viral genes coming from the human MERS (ENc ~ 50.40), followed by bats MERS-like (ENc ~ 49.64). The hedgehogs MERS-like showed an ENc median of 47.70, similar to those of SARS and SARS-CoV-2. Civets SARS-like showed an ENc median of 47.76. For bats SARS-like, the ENc median was 47.80 and the ENc median for the human SARS was 47.66. The lowest ENc values were observed for the genes of SARS-CoV-2-like. SARS-CoV-2-like coming from bats showed a median ENc of 46.39, while the SARS-CoV-2-like coming from pangolins showed an ENc median of 44.80 and for the human SARS-CoV-2, the ENc median was 44.34 (Fig. 1C).

When we classified the viral according to the gene family, we observed that the envelope protein E presented ENc values that ranged from 42 to 61, being the genes of the human SARS-CoV-2 and bat SARS-CoV-2-like the ones that presented the lowest values. The membrane glycoprotein M genes presented ENc values that ranged from 43.32 to 61.00, and the gene of the hedgehogs MERS-like, was the one with the lowest ENc. The membrane glycoprotein M of the human MERS, civets, bats MERS-like, and humans SARS showed virtually non-biased codon usage. The nucleocapsid protein N showed ENc values that ranged from 49.08 to 55.30, being the human MERS gene and the bat MERS-like gene, the viral genes that presented the lowest Enc values.

The genes that encode for the spike protein S presented ENc values that ranged from 44.16 to 47.68. For the human SARS-CoV-2, the spike protein S showed the lowest ENc value, followed by the gene of the SARS-CoV-2-like coming from bats and pangolins. The ORF genes presented ENc values that ranged from 26.60 to 57.89, being the human SARS-CoV-2, the virus that presented the lowest and the highest ENc values for ORF7 and ORF10 respectively.

### CPB analysis between viruses and lung tissue highly expressed genes

**Figure 2:**
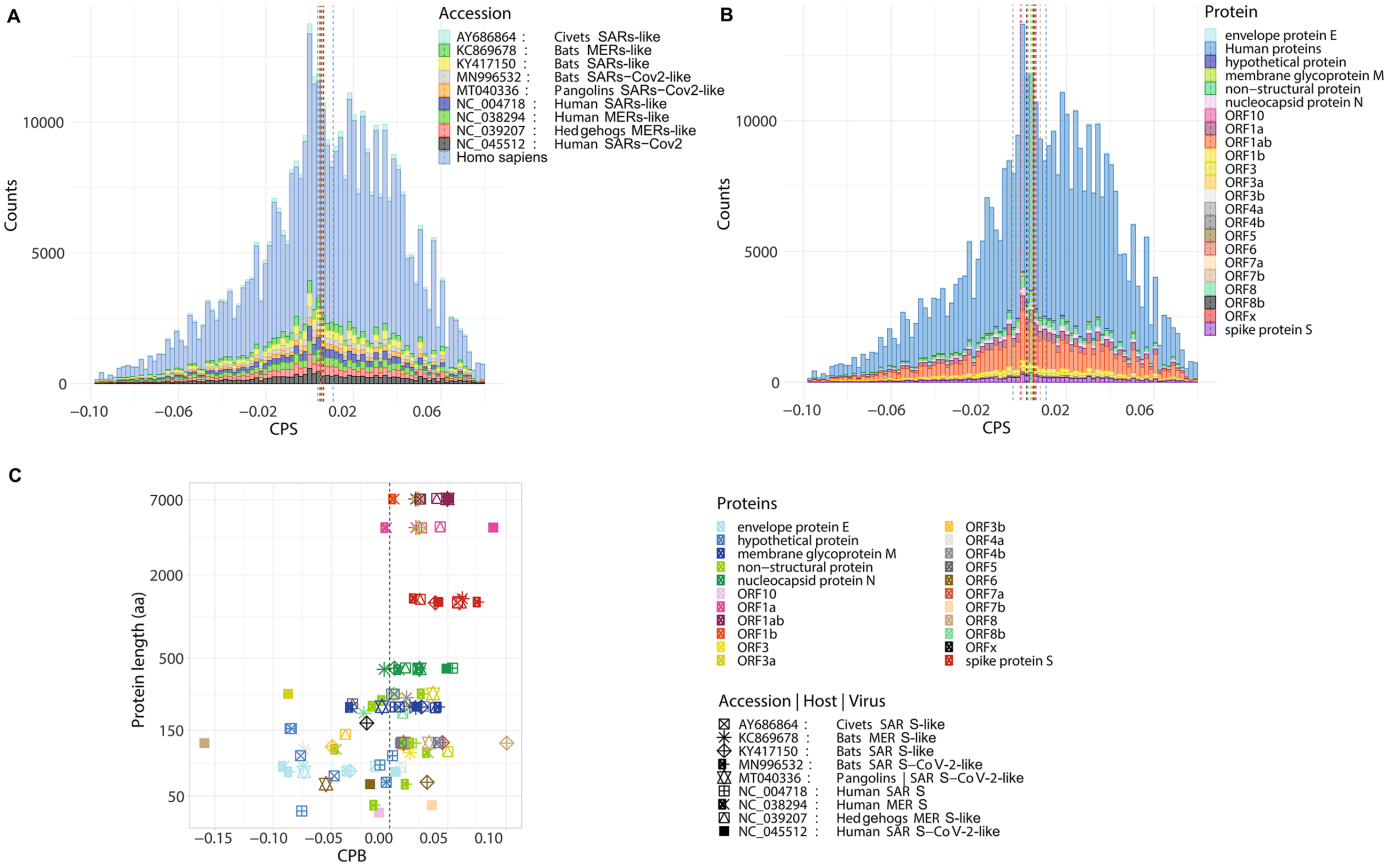
Codon pair score of viral genes of SARS-CoV-2 (NC_045512), SARS (NC_004718) and MERS (NC_038294), and the related virus of non-human hosts: civets SARS-like (AY686864), bats MERS-like (KC869678), bats SARS-like (KY417150), bats SARS-CoV-2-like (MN996532), pangolins SARS-CoV-2-like (MT040336) and hedgehogs MERS-like (NC_039207) and human genes. (**A**) Codon pair frequencies for each virus. (**B**) Codon pair frequencies for each gene and classified by type of viral gene. (**C**) Codon pair bias for each viral gene vs protein length.

Closely related species have a similar CPB. Since viruses replicate exclusively inside the living cells of their hosts, many viruses are influenced by the host codon pair preferences, being the reflection of the CPB or CPS of their hosts. A negative CPS value means that a particular codon pair is underrepresented, whereas a positive CPS value indicates that a particular codon pair is overrepresented in the analysed protein coding sequences. When the viral genes present CPB and CPS values that positively correlate with those of their hosts, this results favourable for the virus viability^34,54^. Here, we evaluated the CPS of the viral genes and compared it with the CPS of the human genes to determine whether the viruses that infect humans have similar codon pair preferences to their hosts. Viral genes showed lower CPS frequencies than human genes. The median CPS for the viral genes was 0.053 for civet SARS-like, 0.051 for bat SARS-like, and 0.053 for human SARS. The median CPS for the MERS genes was 0.048 for the hedgehogs MERS-like, 0.046 for the bats MERS-like and 0.037 for humans MERS. SARS-CoV-2 showed median CPS values of 0.064 for the bat SARS-CoV-2-like, 0.065 for the pangolins SARS-CoV-2-like and 0.061 for humans SARS-CoV-2. The median CPS for the highly expressed human genes in lungs was 0.11 (Fig. 2A). When we classified the viral genes according the gene families, we observed that the ORF7b and spike protein S were the genes with the highest median of CPB values (0.081 and 0.061 respectively), followed by ORF3a and nucleocapsid protein N (0.058 and 0.057) indicating that particular codon pairs are overrepresented in these genes and therefore they have a higher level of optimization. Envelope protein E and the membrane glycoprotein M showed values of 0.048 and 0.044, respectively (Fig. 2B). Furthermore, we observed different CPB values among the viral genes’ families of the different viruses according to the host they infect (Fig. 2C). The bat SARS-like showed the highest CPB for ORF7b (~ 0.1) and ORF7a (~ 0.048). The human SARS-CoV-2 showed the highest CPB values for the ORF1a (0.089), ORF1ab (0.051), the nucleocapsid protein N (0.048) and the spike protein S (0.042). The envelope protein E and the membrane glycoprotein M presented CPB values of 0.0005 and 0.02, respectively. In pangolins SARS-CoV-2-like, the highest CPB value was for the spike protein S (0.060), followed by ORF3a (0.037) and ORF8 (0.033). Whereas for the bats SARS-CoV-2-like, the highest CPB value was for the spike protein S (0.076), followed by ORF1ab (0.050). The highest CPB values for the genes of the human MERS were observed for a non-structural protein (0.032), the spike protein S (0.021), and the nucleocapsid protein N (0.008). In the bat MERS-like, the highest CPB value was for spike protein S (0.062), followed by the ORF1a (0.022) and by the membrane glycoprotein M (0.022). In the hedgehogs MERS-like, the highest CPB value was for the ORF3a (0.049), followed by the ORF1a/b (0.042) and the membrane glycoprotein M (0.029). In order to evaluate the fitness and the specialization of the viruses in their hosts, we compared the CPS of the viral genes against the CPS of the overexpressed human genes in lung tissue by performing CPS correlation analysis. The 3721 codon pairs of all the viruses were compared with the 3721 codon pairs of the human genes. The correlation analysis showed low R values with no clear dependence on the human host codon pairs (Supplementary Information 3). Neither for the viruses coming from the animal hosts, nor for the human viruses.

### Viral genes clustering analysis

The hierarchical clustering of viral genes provided additional qualitative information about how similar certain genes are in terms of their molecular and codon usage features (Supplementary Information 4). All the genes encoding for the viral nucleocapsid protein N of all the viruses grouped together demonstrating a high level of conservation. To gain accuracy and confidence in the clustering we used an iterative bootstrapping method using which means that after a hundred repetitions, the cluster was statistically stable. We also evaluated the statistical significancy of the results using the chi-square independence test.

The envelope protein E genes grouped in different clusters. For the three viruses, civets, bats SARS-like and human SARS, the envelope protein E grouped with those of the bats and pangolins SARS-CoV-2-like and showed high and similar CPB values. Whereas the gene of the humans SARS-CoV-2 grouped with the genes of the humans and bats MERS-like. and showed high CPB values. The hedgehog’s viruses, the envelope protein E gene grouped distantly with ORF and hypothetical proteins. These genes presented a high preference for the codon GCG for Alanine. Furthermore, the codons GGT and CCT were preferable for Glycine and Proline, respectively. The hydrophobicity, CAI, and Fop values were higher for the SARS and SARS-CoV-2 genes. High values of CAI and Fop usually means that the codon usage is optimized for those genes and therefore conduct to a higher expression rate.

The genes encoding for the membrane glycoprotein M genes also appeared in different clusters. For the humans SARS-CoV-2, this gene grouped with those of the human MERS and bats MERS-like. Only the human SARS-CoV-2 gene showed a preference for the codon GCA for Alanine and AGG for Arginine. A preference for the codons CCA, ACT, and GTT for Proline, Threonine and Tyrosine, respectively, was observed. This was the gene that presented the lowest CPB value. The membrane glycoprotein M of the pangolins and bats SARS-CoV-2-like grouped with the genes of the three SARS viruses in a different cluster and showed a higher CPB. The hedgehogs MERS-like grouped distantly. The codon CAC was observed more frequently for Histidine in the three SARS viruses. The pangolins SARS-CoV-2-like showed a preference for GAC for Aspartate and CCA for Proline, whereas the bats SARS-CoV-2-like showed a preference for GGA for Glycine and GTA for Valine.

The genes encoding for the spike protein S appeared in different clusters. For the human SARS-CoV-2, this gene grouped alone with the genes that encode for a hypothetical protein and several ORF proteins. This showed high bias for the codons TTG for Lysine and GAA for Glutamate. The genes for the rest of the viruses clustered together with other ORF genes and with the membrane glycoprotein M of the hedgehogs MERS-like, although distantly. In this cluster, the genes that encode for the ORF genes showed low values of CPB, being the lowest of all the clusters. Conversely, the genes that encode for the spike protein S presented high CPB values. This analysis also showed that the CPB is highly related to the dinucleotide bias.

### Viral and human genes clustering analysis and CPS correlation analysis

Since the viral genes and the total amount of the overexpressed human genes in lung tissue did not show substantial correlation, we performed a clustering analysis using all the viral genes of the human SARS-CoV-2, SARS and MERS and the overexpressed human genes to determine which human genes contribute more to virus fitness specialization and whether it could be dependent on the host translation machinery of only some genes rather than the whole overexpressed human genes in lung tissues (Supplementary Information 1). We also performed control clustering analyses using the under expressed human genes to compare their codon features against the viral genes of the human SARS, SARS-CoV-2 and MERS. Furthermore, the viral genes of bats and pangolins SARS-CoV-2-like, bats and civets SARS-like and bats and hedgehogs MERS-like viruses were also used as control to compare and validate the codon features against those of the overexpressed human genes. These controls were used to validate the clustering results in order to test that the construction of the groups did not occur only by chance.

**Figure 3:**
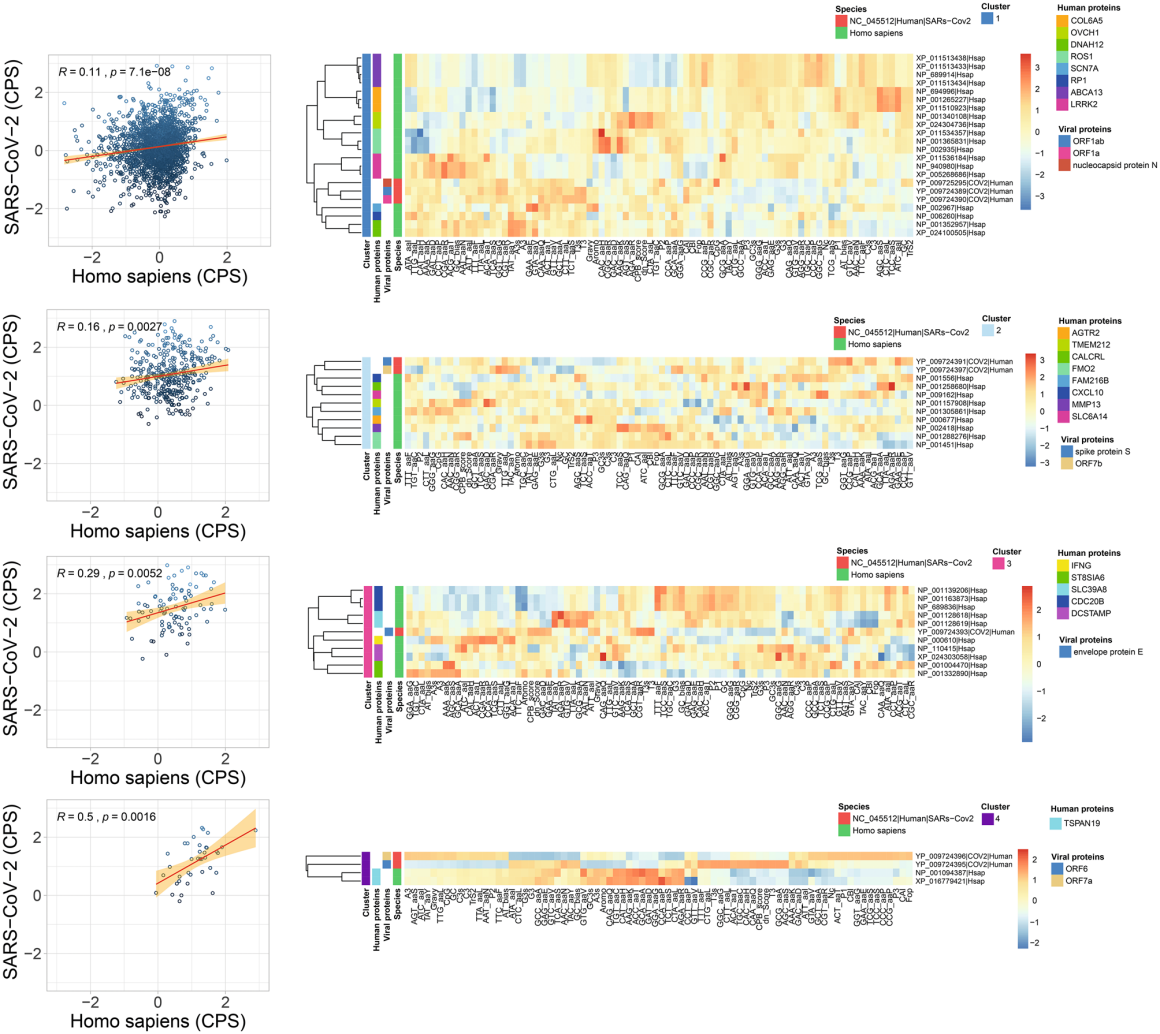
Heatmap of clusters (1–4) using a hierarchical method of viral genes for SARS-CoV-2 (NC_045512) of the human host and human genes based on the molecular features. CPB correlation is included in the left for each cluster relating the CPB of human genes (horizontal axis) and CPB of the viral genes (vertical axis).

From the total 463 highly expressed human genes, 70 genes (15.1%) grouped in clusters together with the viral genes. These were selected and extracted to make an illustrating heatmap (Fig. 3). Furthermore, in order to corroborate whether the viral genes that composed particular clusters present codon pair frequencies that correlate with the human genes of the same clusters, we evaluated the CPS of both, viral and human genes for each block and the correlation between each other.

For the SARS-CoV-2, 8 out of 12 genes grouped with 40 human genes distributed in 4 clusters. The first cluster comprised 3 viral genes: the nucleocapsid protein N, ORF1a and ORF1ab together with 19 human genes: COL6A5 (3), OVCH1 (2), DNAH12 (2), ROS1 (3), SCN7A, RP1, ABCA13 (4) and LRRK2 (3) and the CPS correlation was R⁓0.11 (p-val ⁓ 7.1 × 10^–8^). The CPB values for the human and viral genes were − 0.04 and 0.15, respectively. The second cluster contained 2 viral genes: the spike protein S and ORF7b together with 9 human genes: AGTR2, TMEM212, CALCRL, FMO2 (2), FAM216B, CXCL10, MMP13 and SLC6A14. For this group, the CPS correlation between viral and human genes was R ⁓ 0.21 (p-val ⁓ 0.0027), and the CPB values for the human and viral genes were 0.05 and 0.12 respectively. The third cluster contained only 1 viral gene, the envelope protein E, with 10 human genes: IFNG, ST8SIA6 (2), SLC39A8 (2), CDC20B (3) and DCSTAMP (2), and the CPS correlation between viral and human genes were R ⁓ 0.29 (p-val ⁓ 0.0052) and the CPB values for the human and the viral genes were 0.25 and 0.16 respectively. In the fourth cluster, 2 human genes, TSPAN19 (2) grouped with the viral genes ORF6 and ORF7a and showed a CPS correlation of R ⁓ 0.5 (p-val ⁓ 0.0016). The CPB values for the human and viral genes were 0.13 and 0.14, respectively.

For the SARS virus (Supplementary Information 5), 12 out of 14 genes grouped with 39 human genes distributed in 5 clusters. The first cluster contained 11 human genes: AGTR2, TMEM212, CALCRL, FMO2 (2), FAM216B, CXCL10, MMP13, SLC6A14, and BCL2A1 (2) and 7 viral genes: envelope protein E, hypothetical proteins (5) and membrane glycoprotein M. The CPS correlation for human and viral genes was R ⁓ 0.28 (p-val ⁓ 2 × 10^–16^). The second cluster was composed of 7 human genes: C7orf77, SNTN (2), MUC1 (2) and WIF1 (2). The CPS correlation between human and viral genes was R ⁓ 0.77 (p-val ⁓ 0.00078). The third cluster consisted of 19 human genes:

COL6A5 (3), OVCH1 (2), DNAH12 (2), ROS1 (3), SCN7A, RP1, ABCA13 (4) and LRRK2 (3) and 2 viral genes: ORF1a and ORF1ab. The CPS correlation between human and viral genes was R ⁓ 0.31 (p-val ⁓ 2.2 × 10^–16^). The fourth cluster was composed of 2 TSPAN19 human genes and one viral hypothetical protein. CPS correlation for this cluster was statistically not significant.

**Figure 4:**
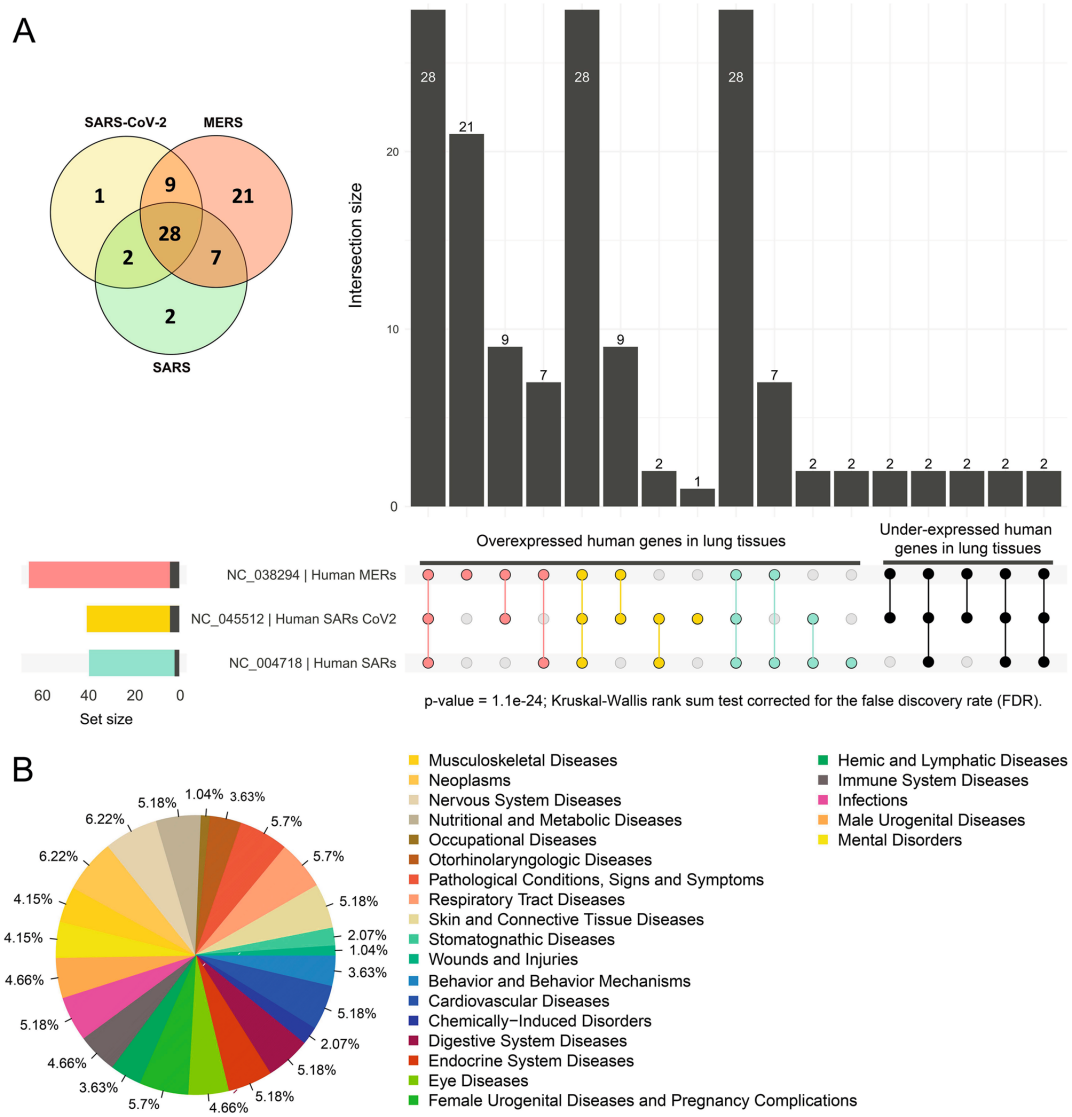
(**A**) Venn diagram and UpSet plot representing the number of overexpressed and under-expressed human genes in lung tissues that clustered together with viral genes for SARS-CoV-2 (NC_045512), SARS (NC_004718) and MERS (NC_038294) based on the molecular features. (**B**) Diseases frequencies associated to human genes grouped with viral genes of SARS-CoV-2, SARS and MERS in the clustering analysis. The differences observed among the groups and gene counts showed to be significant according to the chi-square test that showed a p-value of 1.1e^−24^ (Kruskal–Wallis rank sum test) and corrected for the false discovery rate (FDR).

For the MERS virus (Supplementary Information 6), the 10 genes grouped into 6 clusters together with 65 human genes. The first cluster comprised 19 human genes: COL6A5 (3), OVCH1 (2), DNAH12 (2), ROS1 (3), SCN7A, RP1, ABCA13 (4) and LRRK2 (3) and 2 viral genes: ORF1a and ORF1b. CPS correlation for human and viral genes was R ⁓ 0.29 (p-val ⁓ 0.021). The second cluster was composed of 8 human genes: AGTR2, TMEM212, CALCRL, FMO2 (2), CXCL10, MMP13, and SLC6A14, and 1 viral gene that encodes for the spike protein S. The third cluster contained 6 human genes: CLEC12A (3), FAM216B, DNAAF6, and PIH1D3 and 3 viral genes: non-structural protein (2) and the nucleocapsid protein N. The fourth cluster consisted of 7 human genes: C7orf77, SNTN (2), MUC1 (2) and WIF1 (2) and 1 viral gene encoding for a non-structural protein. The fifth cluster was composed of 9 human genes: ST8SIA6 (2), SLC39A8 (2), CDC20B (3), and DCSTAMP (2) and 2 viral genes: non-structural protein and membrane glycoprotein M. The sixth cluster comprised 16 human genes: CLEC6A, IL1RL1 (2), VNN2 (4), RTKN2 (2), SDR16C5 (3), IL18R1 (2), ACADL, and DNAH12 and the viral gene that encodes for envelope protein E. In contrast, the clustering of the viral genes of the human SARS-CoV-2, SARS and MERS with under-expressed human genes grouped with only 10 human genes. 5 out of 10 for SARS-CoV-2 and MERS and 3 out of 10 for SARS. The differences observed among the groups and gene counts showed to be significant according to the chi-square test that showed a p-value of 1.1e^−24^ (Kruskal–Wallis rank sum test) and corrected for the false discovery rate (FDR) (Fig. 4A).

In addition, in order to corroborate that the clusters of human and viral genes did not occur only by chance, we performed the same clustering analysis as before but as a control we used the viral genes of SARS-CoV-2-like, SARS-like and MERS-like coming from animals (bats, civets, hedgehogs and pangolins) and the 70 overexpressed human genes that had grouped with the viral genes of the human SARS-CoV-2, SARS, and MERS (Supplementary Information 7). The clustering of these human genes with those of the bat SARS-CoV-2-like did not give arise to any group containing viral and human genes together (Supplementary Information 7A). Whereas, with the pangolin SARS-CoV-2-like genes, the clustering showed 2 out of 12 groups that contained human and viral genes. The membrane glycoprotein M and the nucleocapsid protein N grouped with 12 human genes: AGTR2 (1), DCSTAMP (2), FMO2 (2), IFNG (1), MMP13 (1), SLC39A8 (2), ST8SIA6 (2), TMEM212 (1). On the other hand, ORF6 grouped with TSPAN19 (2), (Supplementary Information 7B). The differences observed between the groups of human SARS-CoV-2 and the animal SARS-CoV-2-like with the overexpressed human genes were statistically significant according to the chi-square test (p-value of 1.039e^–15^, Kruskal–Wallis rank sum test and corrected FDR).

The clustering between the human genes and the viral genes of the bat SARS-like showed that only 2 out of 12 clusters contained viral and human genes. The membrane glycoprotein M and the nucleocapsid protein N grouped with 7 human genes: IFNG, AGTR2, TMEM212, CALCRL, FMO2 (2) and MMP13. And the ORF6 grouped with TSPAN19 (2), DNAAF6 and PIH1D3 (Supplementary Information 7C). With respect to the SARS-like coming from the civets, 3 out of 12 clusters contained human and viral genes. One of the clusters grouped the human gene CXCL10 (1) with 5 viral genes: hypothetical protein (3), ORF1ab, and the spike protein S. Other cluster comprised 16 human genes: AGTR2 (1), C7orf77 (1), DCSTAMP (2), FAM216B (1), FMO2 (2), IFNG (1), MMP13 (1), MUC1 (2), SLC39A8 (2), ST8SIA6 (2) and TMEM212 (1) and 2 viral genes: the membrane glycoprotein M and the nucleocapsid protein N. The last cluster comprised 2 human genes: TSPAN19 (2) and a hypothetical viral protein (Supplementary Information 7D). The difference observed among the clusters were statistically significant according to the chi-square test (p-value of 2.35e^–12^, Kruskal–Wallis rank sum test and corrected FDR).

For the bat MERS-like virus 3 out of 12 clusters contained viral and human genes. The ORF4a grouped with 12 human genes: AGTR2 (1), CALCRL (1), DCSTAMP (2), FMO2 (2), MMP13 (1), SLC39A8 (2), ST8SIA6 (2) and TMEM212 (1), The envelope protein E grouped with FAM216B and CXCL10. Whereas the ORF8b grouped with BCL2A1 (2) (Supplementary Information 7E).

Regarding the hedgehogs MERS-like, 3 out of 12 clusters contained human and viral genes. The human gene CXCL10 (1) grouped with 8 viral genes: the membrane glycoprotein M, the nucleocapsid protein N, ORF1a, ORF1ab, ORF3a, ORF4b, ORF5, and the spike protein S. In other cluster the envelope protein E grouped with 3 human genes, the SDR16C5 (3). On the other hand, the viral ORF8b gene grouped with the human gene IFNG (1) (Supplementary Information 7F). These differences among the clusters between animals and human viruses were statistically significant according to the chi-square test (p-value of 6.5e^–13^, Kruskal–Wallis rank sum test and corrected FDR).

These results showed that in all the cases, the number of clusters containing both, overexpressed human genes and viral genes coming from the human SARS-CoV-2, SARS and MERS was higher than in the control clustering using viral genes coming from animal hosts. Moreover, the number of human genes and viral genes from the human SARS-CoV-2, SARS and MERS was also higher than the control. Indeed, for the human SARS-CoV-2 virus, 8 out of 12 genes grouped with 40 human genes, for the human SARS virus, 12 out of 14 genes grouped with 39 human genes and for the human MERS virus, all the 10 viral genes grouped with 65 human genes. Furthermore, we observed that 28 out of 70 human genes comprised a core of genes that appeared into the clusters together with the viral genes for the three human viruses (Fig. 4, Table 1). Between MERS and SARS-CoV-2, only 9 human genes were shared, 7 genes were shared between only MERS and SARS, and 2 genes were shared between only SARS-CoV-2 and SARS. In contrast, 3 viral genes of the pangolin SARS-CoV-2-like grouped with 14 overexpressed human genes and non-gene of the bat SARS-CoV-2-like grouped with any overexpressed human genes. 8 viral genes of the civets SARS-like and 3 of the bat SARS-like grouped with 11 and 19 overexpressed human genes respectively. Furthermore, 10 viral genes of the hedgehog MERS-like and 3 of the bat MERS-like grouped with 5 and 16 overexpressed human genes respectively. The total differences among the groups were statistically evaluated and showed to be significant according to the chi-square test (p-value of 2.9e^–09^, Kruskal–Wallis rank sum test and corrected FDR), (Supplementary Information 7G). This clearly showed that the genes from human viruses share more similarities with the human genes that are overexpressed in human lung tissues. On the other hand, the clustering between the viral genes coming from animal viruses and the under-expressed human genes in lung tissues grouped a lower number of human and viral genes for the three viral species: SARS-CoV-2-like, SARS-like and MERS-like. The differences between the groups of the under-expressed human genes and the viral genes coming from animals and the groups of the under-expressed human genes and the viral genes of the human SARS-CoV-2, SARS and MERS were statistically evaluated and showed not to be significant according to the chi-square test (p-value > 0.05, Kruskal–Wallis rank sum test and corrected FDR) (Supplementary Information 7H).

**Table 1:** Human genes shared among the clusters of the three coronaviruses SARS-CoV-2, SARS and MERS.

Accession Id	Gene name	Proteins
NP_000677	AGTR2	Type-2 angiotensin II receptor
NP_001157908	TMEM212	Transmembrane protein 212
NP_001258680	CALCRL	Calcitonin gene-related peptide type 1 receptor precursor
NP_001265227	COL6A5	Collagen alpha-5(VI) chain isoform 1 precursor
NP_001288276	FMO2	Dimethylaniline monooxygenase
NP_001305861	FAM216B	Protein FAM216B
NP_001340108	OVCH1	Ovochymase-1 precursor
NP_001352957	DNAH12	Dynein heavy chain 12, axonemal isoform 4
NP_001365831	ROS1	Proto-oncogene tyrosine-protein kinase ROS isoform 3 precursor
NP_001451	FMO2	Dimethylaniline monooxygenase
NP_001556	CXCL10	C-X-C motif chemokine 10 precursor
NP_002418	MMP13	Collagenase 3 preproprotein
NP_002935	ROS1	Proto-oncogene tyrosine-protein kinase ROS isoform 1 precursor
NP_002967	SCN7A	Sodium channel protein type 7 subunit alpha
NP_006260	RP1	Oxygen-regulated protein 1 isoform 1
NP_009162	SLC6A14	Sodium- and chloride-dependent neutral and basic amino acid transporter B(0+)
NP_689914	ABCA13	ATP-binding cassette sub-family A member 13
NP_694996	COL6A5	Collagen alpha-5(VI) chain isoform 2 precursor
NP_940980	LRRK2	Leucine-rich repeat serine/threonine-protein kinase 2
XP_005268686	LRRK2	Leucine-rich repeat serine/threonine-protein kinase 2 isoform X1
XP_011510923	COL6A5	Collagen alpha-5(VI) chain isoform X1
XP_011513433	ABCA13	ATP-binding cassette sub-family A member 13 isoform X2
XP_011513434	ABCA13	ATP-binding cassette sub-family A member 13 isoform X3
XP_011513438	ABCA13	ATP-binding cassette sub-family A member 13 isoform X6
XP_011534357	ROS1	Proto-oncogene tyrosine-protein kinase ROS isoform X10
XP_011536184	LRRK2	Leucine-rich repeat serine/threonine-protein kinase 2 isoform X8
XP_024100505	DNAH12	Dynein heavy chain 12, axonemal isoform X2
XP_024304736	OVCH1	Ovochymase-1 isoform X1

In addition, the 28 human genes that appeared in the clusters together with the genes of the 3 human viruses were retrieved against PheGenI (https://www.ncbi.nlm.nih.gov/gap/phegeni/#pgGAP) and DisGeNET (https://www.disgenet.org/) in order to classify the human diseases that appears associated with the malfunction of the genes identified here, as an approach for possible human diseases or collateral effects caused by the viral infections (Supplementary Information 1). According to PheGenI and DisGeNET, 14 out of the 28 genes were associated with 27 diseases. All the diseases appeared in nearly equal proportions. A slightly higher frequency was observed for Nervous System Diseases (12 genes), Neoplasms (12 genes), Respiratory Tract Diseases (11 genes), Pathological Conditions Signs and Symptoms (11 genes), Neonatal Diseases and Abnormalities (11 genes) and Cardiovascular Diseases (10 genes) among others (Fig. 4B).

## Discussion

In humans, coronaviruses cause mainly respiratory tract infections. Previously, six coronaviruses were identified as human-susceptible viruses, being the β-coronaviruses, SARS-CoV and MERS-CoV, responsible for severe and potentially fatal respiratory tract infections^55^. In December 2019 rising pneumonia cases caused by a novel β-coronavirus (SARS-CoV-2) occurred in Wuhan, China. The disease was officially named coronavirus disease 2019 (COVID-19). It was found that the genome sequence of SARS-CoV-2 is 96.2% identical to the bat coronavirus RaTG13, whereas it shares 79.5% of identity to SARS-CoV. It has been proposed that the SARS-CoV-2 may have originated from bats or unknown intermediate hosts that could involve pangolins, crossing the species barrier into humans^56^. Rather, bats are the natural reservoir of a wide variety of coronaviruses, including SARS-CoV-like and MERS-CoV-like viruses^57–59^.

Up to late April, a total of ⁓ 500 SARS-CoV-2 β-coronavirus genomes became available and this number is continuously increasing at an unprecedented rate. In our study, we analysed the coronaviruses from different host species and retrieved the phylogeny to select the best candidate genomes for further analysis of codon usage and molecular relationship with the human host. As previously reported, we found that bats seem to be a common natural host or reservoir for SARS-CoV-2, SARS and MERS viruses since phylogenetic analysis always placed a bat virus close to the human viruses.

The viruses do not synthetize their own tRNAs, therefore, for the viral replication and protein synthesis, the tRNAs availability relies exclusively on the host cell machinery. Thereby, there exists a co-evolution phenomenon between RNA viruses and their hosts' codon usages that shapes the viral CUB^60–62^. However, the viral codon usage evolution is more complex involving mutation pressure, particular DNA/RNA or protein structure and genome size. In many cases viral genes encoding structural proteins have more similar codon usage pattern to the host than other genes^28,29,63^. The codon usage frequency varies significantly among genes within the same organism as well as in viruses. In a multicellular host, viruses are normally restricted to specific organ, tissue, or cell type^29^. Therefore, the matching of the average RSCU of any viruses to their hosts is not enough to explain the virus evolution and predict a target host or even for successful viral infection^29^. Furthermore, the adaptation of viruses that replicate in multiple hosts should involve a trade-off between precise and functional matching to fit the diverse tRNA pools of multiple hosts^64^. Conversely, single host viruses are expected to have specialised to match only their host tRNA repertoire. In many cases the human viruses have CUB that matches highly expressed proteins in the tissues they infect^30,31^ and the CUB tend to be more similar to that of the symptomatic hosts rather than that of the natural hosts, supporting a general deleterious effect of excessive CUB similarity between virus and host^65^.

The internalization of the coronavirus is mediated by the Angiotensin converting enzyme 2 (ACE2). This receptor is necessary for the invasion of the virus into the host cell through the viral spike proteins and the transmembrane Serine Protease 2 (TMPRSS2). The respiratory tract and lungs are the main path that the coronaviruses use to enter the organism and ACE2 is expressed in lung tissue providing the route to the virus cell internalization^31,56,65,66^. Therefore, in our work, we selected the overexpressed genes in lung tissues and compared their codon usage patterns and molecular features with those of the human SARS-CoV-2, SARS and MERS in order to find out which human genes contribute more to the virus fitness and whether the cross-over speciation of the viruses is related with the codon usage similarities of the host. On the other hand, under-expressed human genes and SARS-CoV-2-like, SARS-like and MERS-like isolated from their natural animal hosts were used as controls to evaluate the confidence of our findings. Here we found that all the human viruses had a similar CUB, however the ENc average differs by ⁓ 1 unit from the viruses that come from the animal hosts, reflecting the molecular features of their original host. Furthermore, as demonstrated in our clustering analysis, codon pair usage seemed to be dependent on the dinucleotide bias. The CPB was higher for human genes than for the viral genes as previously reported^67,68^. Positive values of CPB indicate that a particular codon pair is overrepresented and that the level of optimization is higher. Furthermore, different viral gene families exhibited different CPB values, which demonstrates that different kinds of viral genes have different levels of optimization. Our analyses also allowed us to distinguish the main factors that contribute to the genes distribution in the PCA that are a reflection of the molecular differences among the viral genes. The genes of human and non-human viruses showed some differences that could be important for explaining the virus infection evolution. The most important pattern seems to be the A/T content in the third codon position. The genes of the human SARS-CoV-2 distributed away from genes of the bats and pangolins SARS-CoV-2-like, being the A/T content the main factor that contributed to the dispersion, which is also in accordance with previous reports^67,69–71^. Human genes present a wide rate of GC content (27–97%) and the use of A/T ending codons is also a common feature in several human genes^72,73^. The clustering analyses also revealed similar features between the viral genes of human viruses and some of the overexpressed human genes in lung tissue. Conversely, the level of similarity between under-expressed human genes and those of SARS-CoV-2, SARS and MERS as well as the viral genes of the viruses coming from animal hosts and the overexpressed human genes was remarkably lower. This suggests that the viruses would have mutated, increasing the level of similarity with human codon usage patterns. Thus, they could have been favoured for the adaptation to the human hosts translation machinery. The case of SARS-CoV-2 is quite striking because despite the genome sequence of the bat SARS-CoV-2-like is more similar to the human SARS-CoV-2, when we moved from bats to pangolin and then to humans, we observed an increasing number of viral and human genes that cluster together. The set of molecular features and the variability that intervene for and against grouping human and viral genes is complex. Not all the overexpressed human genes share the same features with each other, therefore some features become more relevant to define the final groups. The main differences could be explained by a higher dispersion within the genes of the pangolin SARS-CoV-2-like, whose genes, in some cases, share codons such as CCA for Proline, TAC for Tyrosine, CPB, translational selection (TrS2), CBI, Fop and low aromaticity permit to group human and viral genes. Others have a higher CU of CGA for Glutamine, TCT for Serine, AAT for Asparagine, GTT for Leucine. All of them presenting a high frequency of T in the last codon position^29^. In these genes, a higher hydrophobicity and aromaticity also play a higher role. Therefore, if the cross-over speciation from species to species happened, these results suggest that it could have occurred from pangolins to humans as previously suggested^74–76^ or in a coexistent of both niches bats and pangolin cross-over in close relation with humans. The results of the clustering analysis also suggested that important viral genes such as the membrane glycoprotein M, that is involved in the membrane transport of nutrients, the bud release, the formation of the envelope, the virus assembly and in the biosynthesis of new virus particles^56^, distributed differentially from the non-human viruses indicating that it is highly influenced by A/T content. Surprisingly, this gene was positioned near the hedgehog’s MERS-like gene, suggesting similar molecular patterns between two distant viruses. The envelope protein E, that functions as an ion channel and regulates the virion assembly and the immune system of the host^55,56^, showed the same tendency toward A/T ending codons for the human viruses SARS-CoV-2 and MERS. Both, membrane glycoprotein M and envelope protein E genes have a higher CUB in comparison with human MERS and SARS, which is not in accordance with the trade-off theory that postulates that cross-species virus transmission demands relaxing the codon usage pattern^68^. However, this phenomenon could be explained by a selection pressure in favour of the virus replication in the new host or due to the recent cross-species virus transmission as we know it has occurred. The analysis of new isolates from infected humans through the time presented a higher variance (data not shown) for those genes demonstrating that different stains of the virus coexist within the population and suggesting that both phenomena could be occurring. Nevertheless, only the envelope protein E clustered together with human genes, demonstrating similar molecular patterns that could mean an advantage for virus replication in humans, facilitating the virion assembly and the regulation of the immune system in humans. Furthermore, positive CPB and an incremented CPS correlation for the cluster that grouped the envelope protein E with human genes supports the hypothesis of a facilitated translation depending on codon usage and codon pairs. Similar patterns were observed for ORF6 and ORF8 genes, which are involved in the viral pathogenesis, apoptosis induction, and inflammatory responses in the host^1,77^. These genes grouped with human genes in different clusters and showed also and incremented CPS correlation.

Studies in different viruses species have reported that the nucleocapsid protein N is highly conserved through the virus families^68,78–81^. Therefore, as expected, the codon usage and molecular features presented a similar pattern for all the coronaviruses. Nevertheless, the nucleocapsid gene of the human SARS-CoV-2 tended to distribute slightly far from the rest of viruses’ capsids genes and toward a higher A/T content in the PCA. The human SARS and SARS-CoV-2 nucleocapsid protein N present a high CPB suggesting a specialization acquirement in the human host. However, SARS nucleocapsid gene did not group with human genes while SARS-CoV-2 and MERS nucleocapsid did. This indicate that the nucleocapsid protein N of SARS-CoV-2 shares features with those of SARS and MERS but that the translation of SARS-CoV-2 and MERS nucleocapsid protein N could be facilitated in humans. Two viral genes that also presented a high CPB were the ORF1a/b, that encodes for the replicase complex (polyproteins pp1a and pp1ab) and the Spike protein S that participates in the early viral infection by attaching to the host receptor ACE2 and mediating the internalization of the virus^56^. The ORF1a/b grouped with the gene that encodes for the nucleocapsid protein N, indicating that their molecular features are also conserved. This result is in concordance with previous works that proposed these genes as well as the protein S as candidates for deoptimization for the design of attenuated vaccines due to their high positive CPB values^67^. Nevertheless, the gene that encodes for the spike protein S that also presents high and similar positive CPB values, grouped with ORF7 that is involved in viral pathogenesis and apoptosis induction. All of them showed a high rate of A/T content in the third codon position. Changes in the third codon position produce synonymous substitutions that could have conducted to a codon optimization in human cells and could be further benefited by the host machinery that translates those genes whose molecular features match the viral needs. Thus, some viral genes could be favoured for an increased viral replication in humans and optimized by using or mimicking the molecular patterns of some human genes that are overexpressed in the lung tissue. Indeed, as demonstrated by the clustering results, more viral and human genes grouped together when we used the viral genes of the humans SARS-CoV-2, SARS and MERS. Instead, when we used as a control the viral genes of viruses coming from animal hosts, the number of genes that grouped together was remarkably lower. This suggests that the human viruses would have undergone a kind of optimization in humans with respect to the viruses coming from animal hosts that were used as controls. However, only some genes, such as the envelope E, the ORF 6 and 8, could be the key for an exacerbated viral pathogenesis. Furthermore, due to the high molecular and codon usage similarities between some overexpressed human and some viral genes, the translation machinery of the host could propitiate the translation of viral genes to the detriment of human gene expression of those genes in lung tissues. Indeed, mistranslation or deregulation of protein synthesis has been reported as a consequence of the tRNA miss-modification and imbalanced tRNA expression, conducting to diseases^82^. Recent studies have also proposed that an unbalance in the tRNAs pools of the infected cells could occur and would explain the collateral effects observed in some viral infections^70^. Studies using Ribo-Seq datasets from virus-infected yeast and human cells have shown that viruses CUB trans-regulate tRNA availability, and therefore interfere in the decoding time of the codons having an immediate impact in the protein translation. Furthermore, the virus CUB in symptomatic hosts tended to be more similar than that of natural hosts, supporting a general deleterious effect of excessive CUB similarity between virus and host^65^.

Since COVID-19 outbreak, several pathologies have been associated with COVID-19 and new studies are being performed in order to find out how damaging this new virus is for the human being and which organs or tissues may be infected. As a result of our thorough molecular and clustering analysis, we obtained a list of core human genes that grouped with those of the three human viruses studied here as a consequence of their high molecular similarities. Hereby, if an unbalance in the tRNAs pools occurs due to this high similarity, the translation of these genes could be particularly affected. The malfunction of these genes has been associated with different human pathologies and increasingly more pathologies appear because of the infections with COVID-19. Patients infected with COVID-19 typically present fever and respiratory symptoms. Nevertheless, it has been reported that the risk of complications of hypertension, congestive heart failure, and atherosclerosis conduct to an increased rate of cardiovascular comorbidities^83–85^. Other patients have experimented gastrointestinal manifestations^86^, neurologic complications^87,88^, and complications associated with the endocrine and urogenital systems, among others^89,90^. The genetic component of the virus as well as that of infected individuals associated with the different pathologies and the severity of the infections is still unknown. The high A/T content in the third codon position as well as other shared features between viral and human genes could be the key for a facilitated translation of the viral genes and therefore for its replication. The same molecular and codon usage features that contribute to the formation of the clusters that grouped the viral and human genes could trans-regulate the host tRNA availability conducting to different comorbidities depending on the genetic composition of the human genes. Thus, the genetic population variability may have a role in the development of the disease and collateral effects caused by COVID-19 as a consequence of the malfunction of the genes listed in our work when the codon usage and molecular similarity with the viral genes are high and match the viral needs.

Clearly, other factors such as the ACE2 level expression in different tissues or the biochemical and the immunology responses play important roles in developing diseases. However, this is the first codon usage approach that reveals which genes could be potentially deregulated due to the codon usage similarities between the host and the viral genes when the virus is already inside the human cells of the lung tissue, that is the main route that virus use to invade humans. Our work leads to the identification of additional highly expressed human genes which are not the usual suspects but might play a role in the viral infection and could explain other symptoms in the infected individuals. To identify the genes that could be deregulated under a viral infection is important to determine which individuals would be more susceptible based on their genetic features and comorbidities associated as well as to predict the collateral effects that could appear. Further assays need to be performed in order to truly probe that CU similarities are able to cause this phenomenon under coronavirus infection conditions. Hereby, the genes listed here should be considered to be incorporated into susceptibility population studies for respiratory viral infections. Hereby, these results lay the groundwork for further research in the field of human genetics associated with the new viral infection, COVID-19, caused by SARS-CoV-2 and for the development of antiviral preventive methods.

## Conclusions

In our study, we described the main factors that shape CUB in SARS-CoV-2, SARS and MERS in comparison with highly expressed genes in human lung tissue and revealed matching features with human genes that could have favoured the virus for an incremented pathogenesis. Furthermore, we provided a list of candidate human genes that could be involved in the viral infection and had not been described yet. These genes could be the key for explaining collateral effects and the human susceptibility to viral infections. To identify the human genes that could be deregulated under a viral infection is important to predict the collateral effects and determine which individuals would be more susceptible based on their genetic features and comorbidities associated.

## Supplementary Information

Supplementary Information 1.

Supplementary Information 2.

Supplementary Information 3.

Supplementary Information 4.

Supplementary Information 5.

Supplementary Information 6.

Supplementary Information 7.
