# Supplementary material for: Molecular features similarities between SARS-CoV-2, SARS, MERS and key human genes could favour the viral infections and trigger collateral effects
Source: Sci Rep. 2021 Feb 18;11:4108. doi: 10.1038/s41598-021-83595-1 (PMC7893037; doi:10.1038/s41598-021-83595-1)
Supplement: Supplementary file 2 — Supplementary Information 2. [file 41598_2021_83595_MOESM2_ESM.pdf]

Molecular Features Similarities Between SARS-CoV-2, SARS, MERS and Key Human Genes Could Favour The Viral Infections and Trigger Collateral Effects

Authors: Lucas L. Maldonado*; Andrea Mendoza Bertelli and Laura Kamenetzky

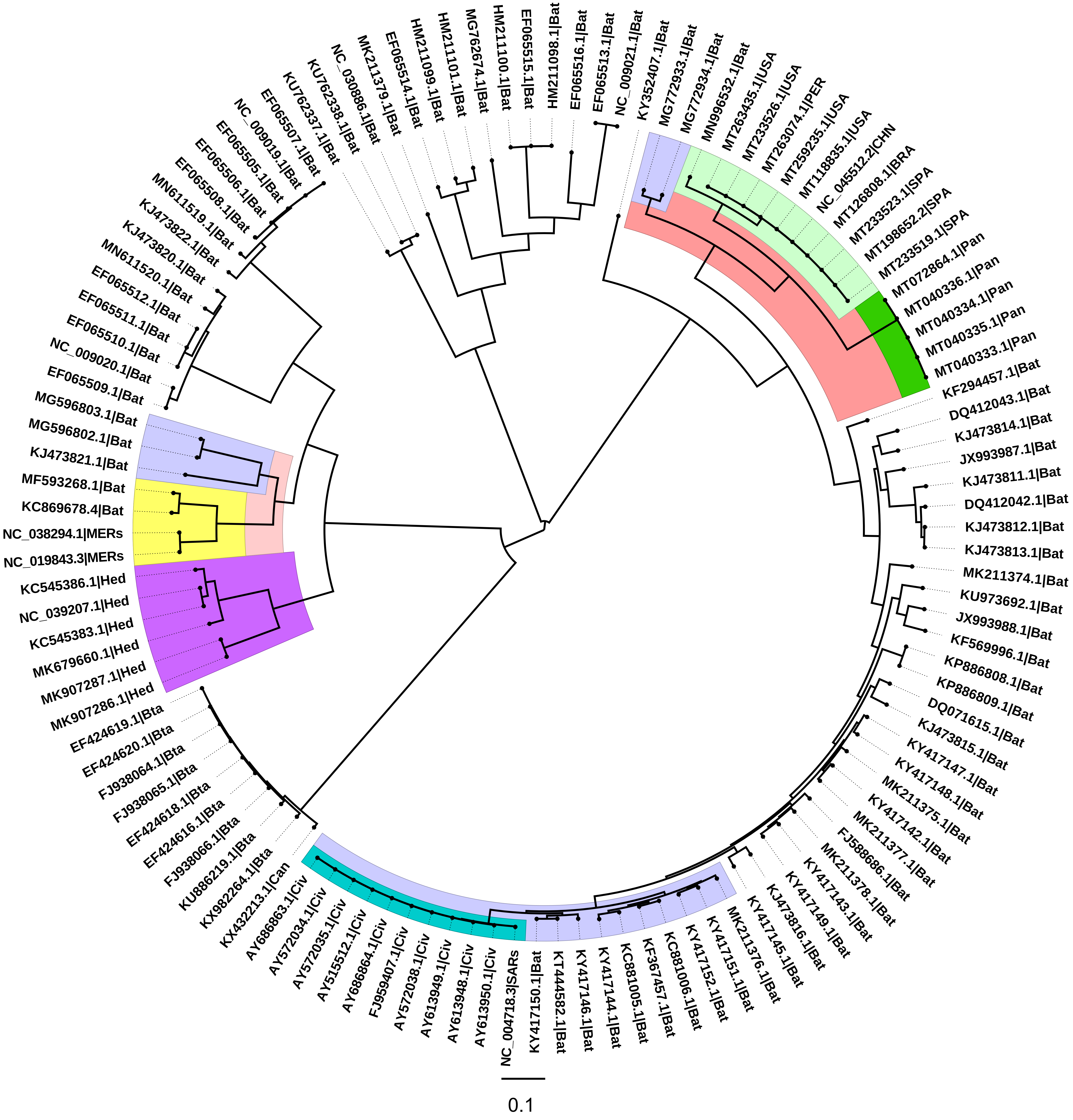

Supplementary file 2: Phylogenetic tree using 118 virus genomes including the references SARS-CoV-2, SARS, and MERS and related viruses belonging to non-human host as described in Supplementary file 1. The clusters were virus grouped with SARS-CoV-2, SARS, and MERS are highlighted. The accession id is followed by the host that the sample was isolated from or the country in the case of different isolations of SARS-CoV-2. Civ: civets, Bta: *Bos taurus*, Hed: hedgehogs, Pan: pangolins.
