# Supplementary material for: Molecular features similarities between SARS-CoV-2, SARS, MERS and key human genes could favour the viral infections and trigger collateral effects
Source: Sci Rep. 2021 Feb 18;11:4108. doi: 10.1038/s41598-021-83595-1 (PMC7893037; doi:10.1038/s41598-021-83595-1)
Supplement: Supplementary file 3 — Supplementary Information 3. [file 41598_2021_83595_MOESM3_ESM.pdf]

Molecular Features Similarities Between SARS-CoV-2, SARS, MERS and Key Human Genes Could Favour The Viral Infections and Trigger Collateral Effects

Authors: Lucas L. Maldonado*; Andrea Mendoza Bertelli and Laura Kamenetzky

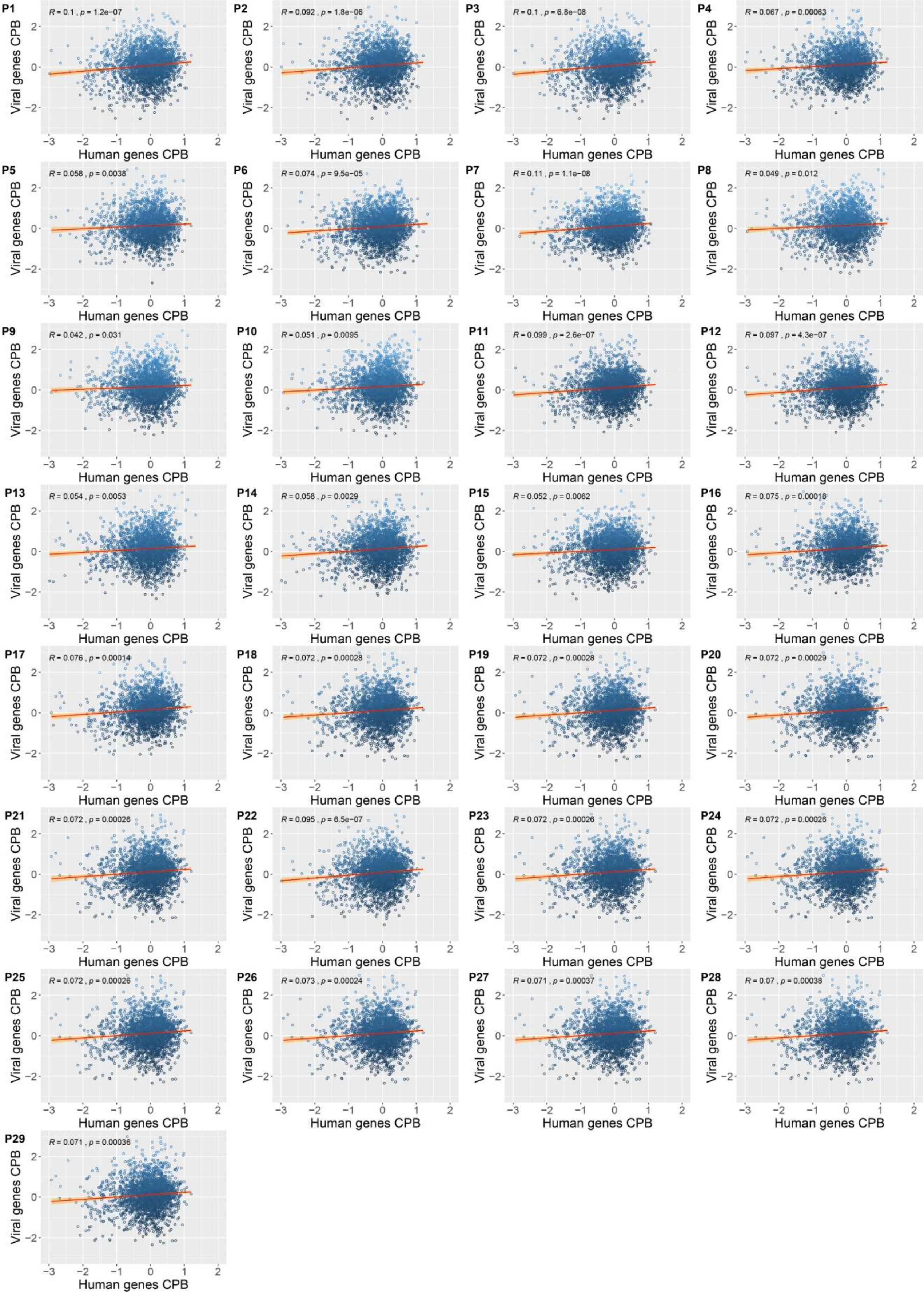

Supplementary file 3: Codon pair bias plots and correlation of viral genes against highly expressed human genes in lungs tissues according to the fold-change between the expression level in lung and the tissue with second highest expression level according to "Human Protein Atlas" (https://www.proteinatlas.org/humanproteome/tissue/lung). P1: KT444582, P2: KY417146, P3: KY417150, P4: MG772934, P5: MN996532, P6: C869678, P7: MF593268, P8: FJ938064, P9: FJ938066, P10: KX432213, P11: Y572035, P12: AY686864, P13: MK679660, P14: NC_039207, P15: NC_038294, P16: T040336, P17: MT040333, P18: MT126808, P19: NC_045512, P20: MT066156, P21: T263074, P22: NC_004718, P23: MT198652, P24: MT233519, P25: MT233523, P26: T118835, P27: MT233526, P28: MT259235, P29: MT263435.
