# Supplementary material for: Molecular features similarities between SARS-CoV-2, SARS, MERS and key human genes could favour the viral infections and trigger collateral effects
Source: Sci Rep. 2021 Feb 18;11:4108. doi: 10.1038/s41598-021-83595-1 (PMC7893037; doi:10.1038/s41598-021-83595-1)
Supplement: Supplementary file 4 — Supplementary Information 4. [file 41598_2021_83595_MOESM4_ESM.pdf]

Molecular Features Similarities Between SARS-CoV-2, SARS, MERS and Key Human Genes Could Favour The Viral Infections and Trigger Collateral Effects

Authors: Lucas L. Maldonado*; Andrea Mendoza Bertelli and Laura Kamenetzky

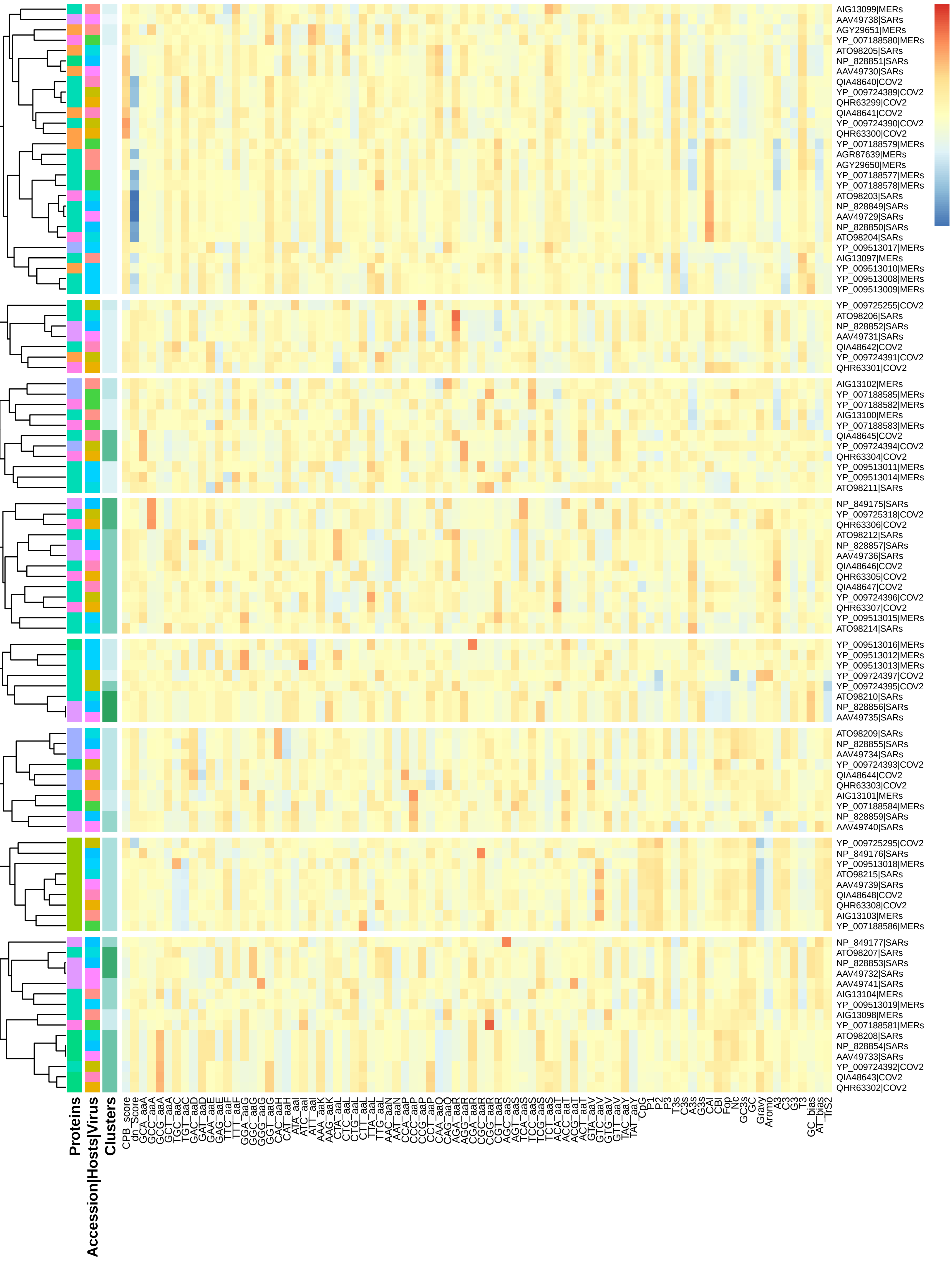

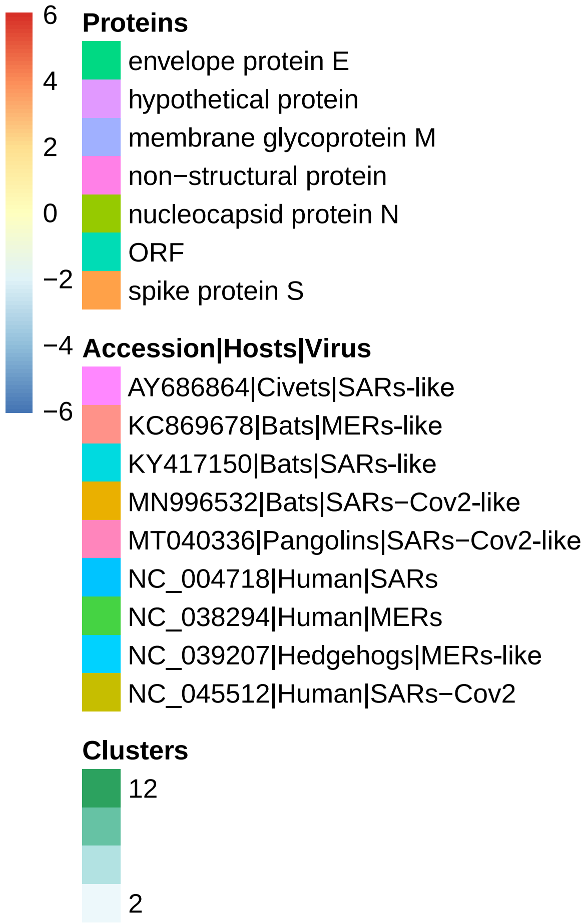

Supplementary file 4: Heatmaps clustering using a hierarchical method of viral genes based on the measures of molecular and codon usage patterns. The viruses whose genes were included here were SARS-Cov-2, SARS, and MERS and the closest viruses to the common nodes in the phylogenetic tree of supplementary file 2. Civets SARS-like (AY686864), bats MERS-like (KC869678), bats SARS-like (KY417150), bats SARS-CoV-2-like (MN996532), pangolins SARS-CoV-2-like (MT040336), human SARS (NC_004718), human MERS (NC_038294), hedgehogs MERS-like (NC_039207) and the human SARS-CoV-2 (NC_045512).
