# Supplementary material for: Molecular features similarities between SARS-CoV-2, SARS, MERS and key human genes could favour the viral infections and trigger collateral effects
Source: Sci Rep. 2021 Feb 18;11:4108. doi: 10.1038/s41598-021-83595-1 (PMC7893037; doi:10.1038/s41598-021-83595-1)
Supplement: Supplementary file 7 — Supplementary Information 7. [file 41598_2021_83595_MOESM7_ESM.pdf]

Molecular Features Similarities Between SARS-CoV-2, SARS, MERS and Key Human Genes Could Favour The Viral Infections and Trigger Collateral Effects

Authors: Lucas L. Maldonado*; Andrea Mendoza Bertelli and Laura Kamenetzky

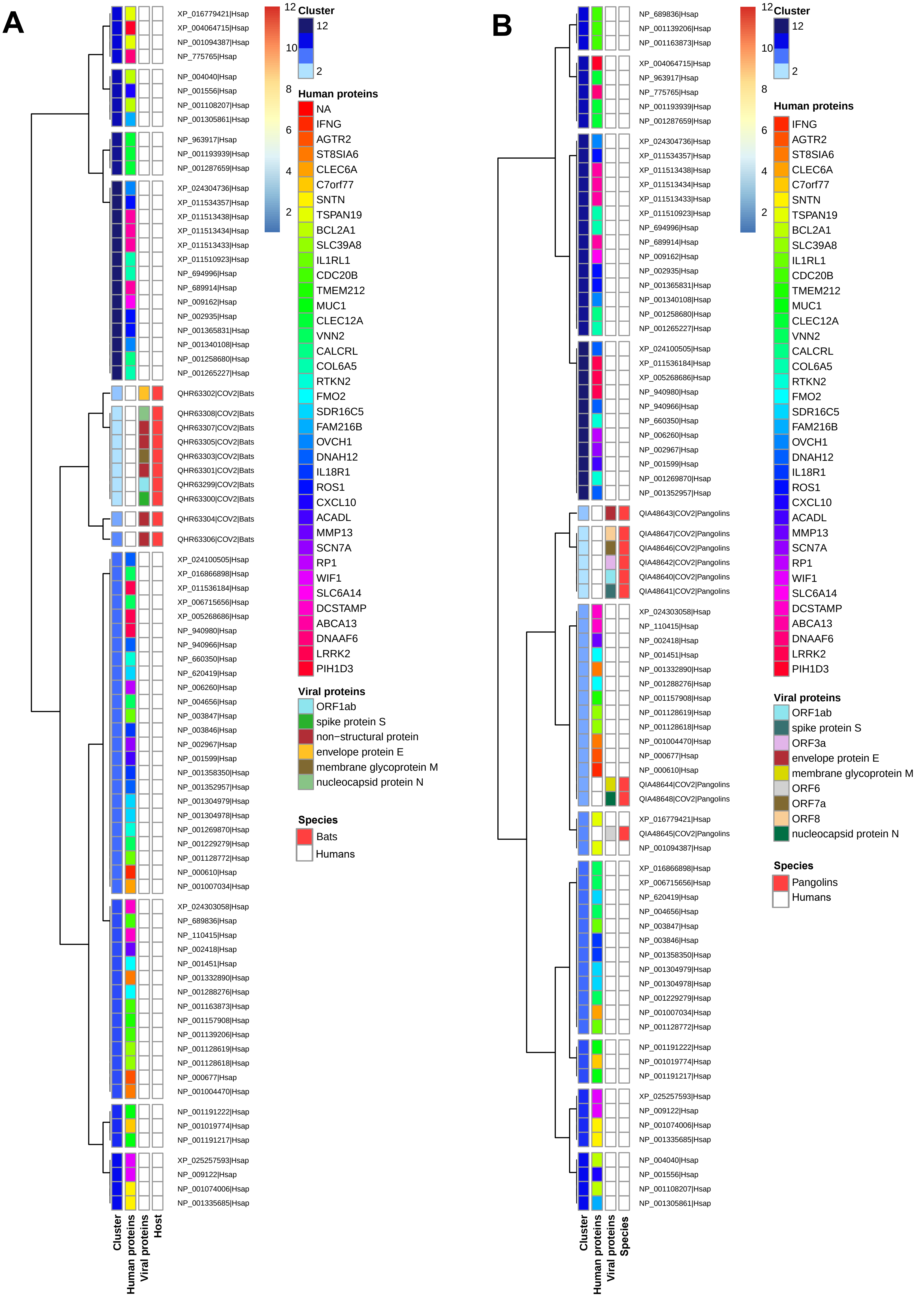

Supplementary file 7: Control clustering based on the molecular features and codon usage patterns using the viral genes of the viruses coming from their animal hosts (bats, pangolins, civets and hedgehogs) and the 70 overexpressed human genes of lungs tissue that grouped with the viral genes of the humans SARS-CoV- 2, SARS and MERS using a hierarchical method and bootstrapping. A). gene clustering of the bat virus SARs-CoV-2-like (MN996532) and 70 overexpressed human genes of lungs tissue. B). gene clustering of the pangolin virus SARS-CoV-2-like (MT040336) and 70 overexpressed human genes of lungs tissue. C). gene clustering of the bat virus SARS-like (KY417150) and 70 overexpressed human genes of lungs tissue. D). gene clustering of the civets virus SARS-like (AY686864) and 70 overexpressed human genes of lungs tissue. E). gene clustering of the bat virus MERs-like (KC869678) and 70 overexpressed human genes of lungs tissue. F). gene clustering of the hedgehogs virus MERS-like (NC_039207) and 70 overexpressed human genes of lungs tissue. G.i). Overexpressed human genes of lung tissues that grouped with SARS-CoV-2, SARS and MERS of humans and animals. Significant differences of groups and counts of human genes are showed according to human and animal hosts in colour or black. The differences observed were statistically tested using the chi-square test. G.ii). Gene clustering of SARS-CoV-2 and under-expressed human genes in lung tissues. G.iii). Gene clustering of SARS and under-expressed human genes in lung tissues. G.iv). Gene clustering of MERS and under-expressed human genes in lung tissues.

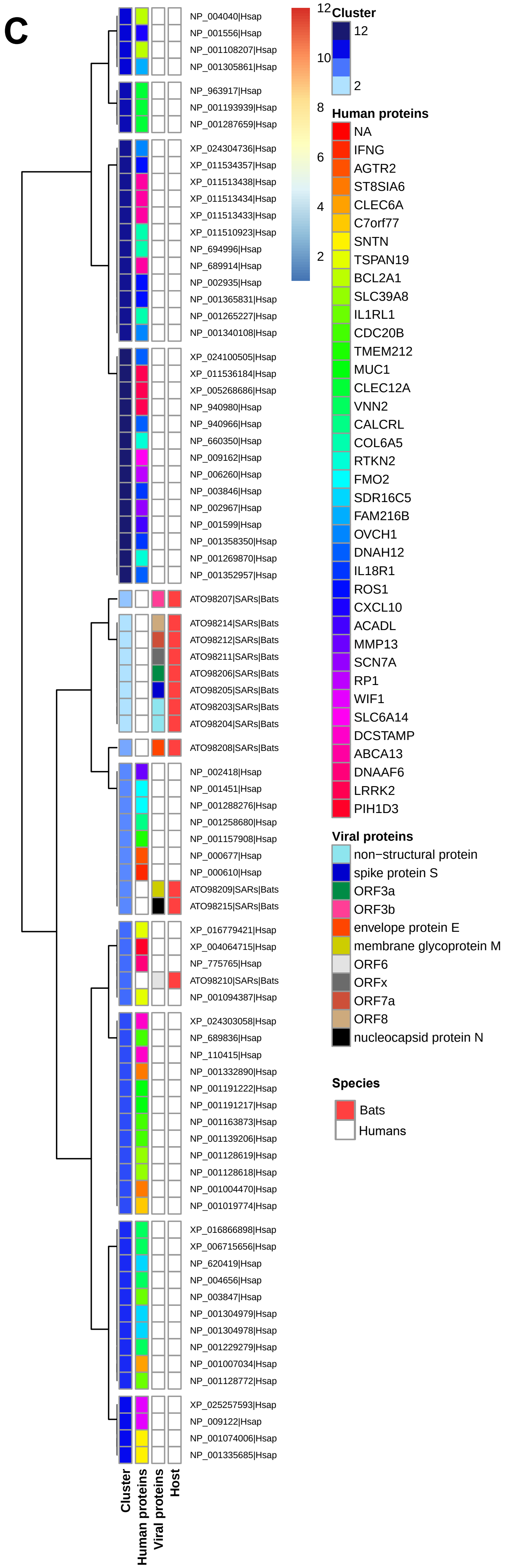

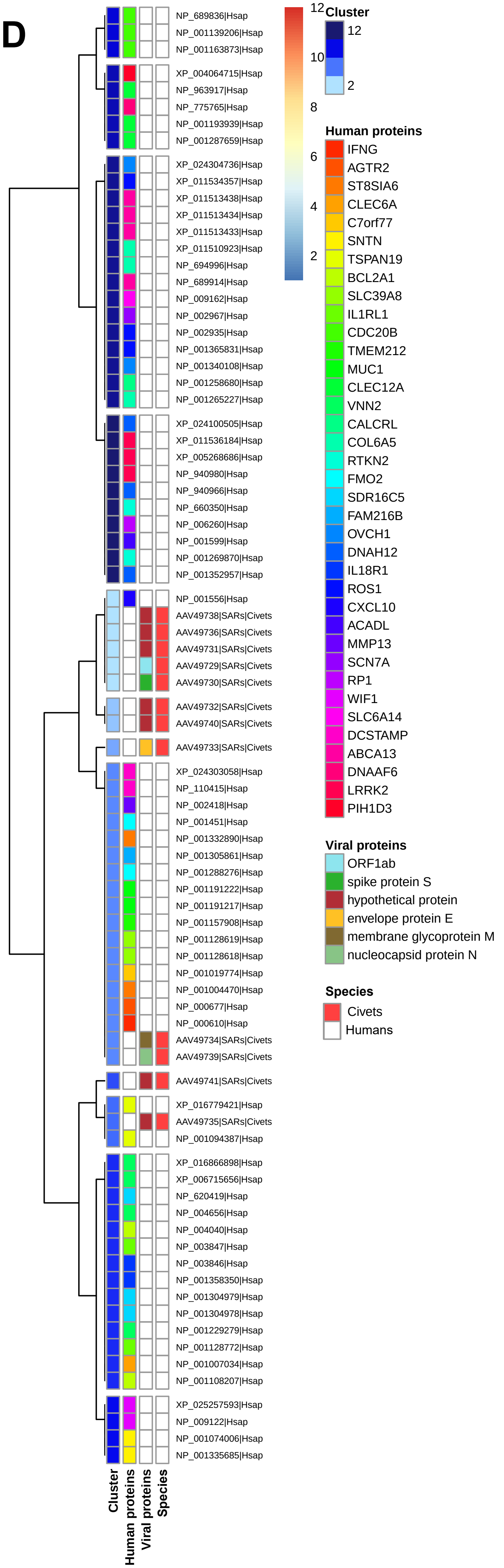

H.i). under-expressed human genes of lung tissues that grouped with SARS-CoV-2, SARS and MERS of humans and animals. Significant differences of groups and counts of human genes are showed according to human and animal hosts in colour or black. The differences observed were statistically tested using the chi-square test. H.ii). gene clustering of the bat virus SARs-CoV-2-like (MN996532) and 66 under-expressed human genes of lungs tissue. H.iii). gene clustering of the pangolin virus SARS-CoV-2-like (MT040336) and 66 under-expressed human genes of lungs tissue. H.iv). gene clustering of the bat virus SARS-like (KY417150) and 66 under-expressed human genes of lungs tissue. H.v). gene clustering of the civets virus SARS-like (AY686864) and 66 under-expressed human genes of lungs tissue. H.vi). gene clustering of the bat virus MERs-like (KC869678) and 66 under-expressed human genes of lungs tissue. H.vii). gene clustering of the hedgehogs virus MERS-like (NC_039207) and 66 under-expressed human genes of lungs tissue.

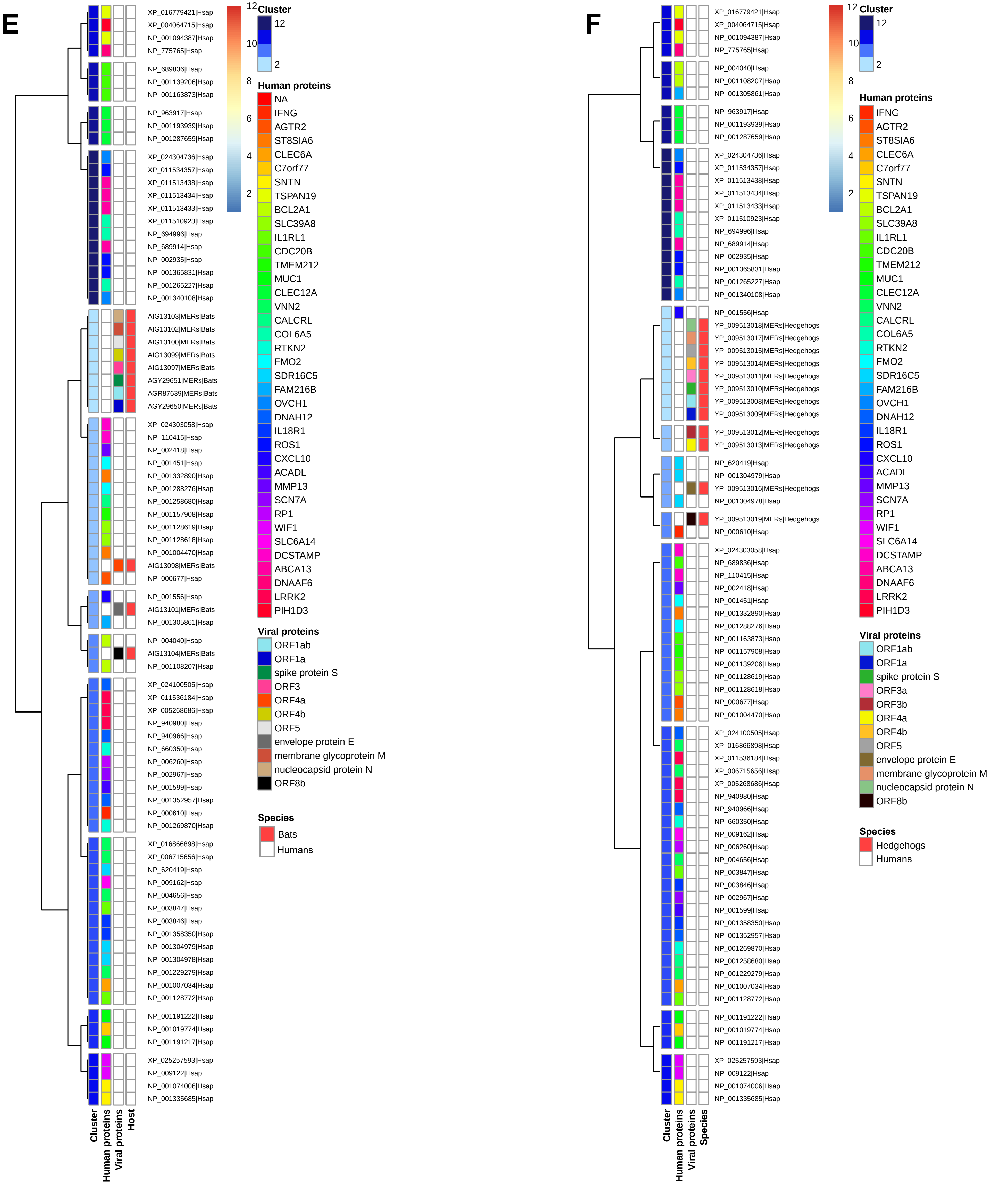
E
XP_016779421|Hsap
XP_004064715|Hsap
NP_001094387|Hsap
NP_775765|Hsap
NP_689836|Hsap
NP_001139206|Hsap
NP_001163873|Hsap
NP_963917|Hsap
NP_001193939|Hsap
NP_001287659|Hsap
XP_024304736|Hsap
XP_011534357|Hsap
XP_011513438|Hsap
XP_011513434|Hsap
XP_011513433|Hsap
XP_011510923|Hsap
NP_694996|Hsap
NP_689914|Hsap
NP_002935|Hsap
NP_001365831|Hsap
NP_001265227|Hsap
NP_001340108|Hsap
AIG13103|MERs|Bats
AIG13102|MERs|Bats
AIG13100|MERs|Bats
AIG13099|MERs|Bats
AIG13097|MERs|Bats
AGY29651|MERs|Bats
AGR87639|MERs|Bats
AGY29650|MERs|Bats
XP_024303058|Hsap
NP_110415|Hsap
NP_002418|Hsap
NP_001451|Hsap
NP_001332890|Hsap
NP_001288276|Hsap
NP_001258680|Hsap
NP_001157908|Hsap
NP_001128619|Hsap
NP_001128618|Hsap
NP_001004470|Hsap
AIG13098|MERs|Bats
NP_000677|Hsap
NP_001556|Hsap
AIG13101|MERs|Bats
NP_001305861|Hsap
NP_004040|Hsap
AIG13104|MERs|Bats
NP_001108207|Hsap
XP_024100505|Hsap
XP_011536184|Hsap
XP_005268686|Hsap
NP_940980|Hsap
NP_940966|Hsap
NP_660350|Hsap
NP_006260|Hsap
NP_002967|Hsap
NP_001599|Hsap
NP_001352957|Hsap
NP_000610|Hsap
NP_001269870|Hsap
XP_016866898|Hsap
XP_006715656|Hsap
NP_620419|Hsap
NP_009162|Hsap
NP_004656|Hsap
NP_003847|Hsap
NP_003846|Hsap
NP_001358350|Hsap
NP_001304979|Hsap
NP_001304978|Hsap
NP_001229279|Hsap
NP_001007034|Hsap
NP_001128772|Hsap
NP_001191222|Hsap
NP_001019774|Hsap
NP_001191217|Hsap
XP_025257593|Hsap
NP_009122|Hsap
NP_001074006|Hsap
NP_001335685|Hsap
Cluster
Human proteins
Viral proteins
Host
12
10
8
6
4
2
Cluster
12
2
Human proteins
NA
IFNG
AGTR2
ST8SIA6
CLEC6A
C7orf77
SNTN
TSPAN19
BCL2A1
SLC39A8
IL1RL1
CDC20B
TMEM212
MUC1
CLEC12A
VNN2
CALCRL
COL6A5
RTKN2
FMO2
SDR16C5
FAM216B
OVCH1
DNAH12
IL18R1
ROS1
CXCL10
ACADL
MMP13
SCN7A
RP1
WIF1
SLC6A14
DCSTAMP
ABCA13
DNAAF6
LRRK2
PIH1D3
Viral proteins
ORF1ab
ORF1a
spike protein S
ORF3
ORF4a
ORF4b
ORF5
envelope protein E
membrane glycoprotein M
nucleocapsid protein N
ORF8b
Species
Bats
Humans
F
XP_016779421|Hsap
XP_004064715|Hsap
NP_001094387|Hsap
NP_775765|Hsap
NP_004040|Hsap
NP_001108207|Hsap
NP_001305861|Hsap
NP_963917|Hsap
NP_001193939|Hsap
NP_001287659|Hsap
XP_024304736|Hsap
XP_011534357|Hsap
XP_011513438|Hsap
XP_011513434|Hsap
XP_011513433|Hsap
XP_011510923|Hsap
NP_694996|Hsap
NP_689914|Hsap
NP_002935|Hsap
NP_001365831|Hsap
NP_001265227|Hsap
NP_001340108|Hsap
NP_001556|Hsap
YP_009513018|MERs|Hedgehogs
YP_009513017|MERs|Hedgehogs
YP_009513015|MERs|Hedgehogs
YP_009513014|MERs|Hedgehogs
YP_009513011|MERs|Hedgehogs
YP_009513010|MERs|Hedgehogs
YP_009513008|MERs|Hedgehogs
YP_009513009|MERs|Hedgehogs
YP_009513012|MERs|Hedgehogs
YP_009513013|MERs|Hedgehogs
NP_620419|Hsap
NP_001304979|Hsap
YP_009513016|MERs|Hedgehogs
NP_001304978|Hsap
YP_009513019|MERs|Hedgehogs
NP_000610|Hsap
XP_024303058|Hsap
NP_689836|Hsap
NP_110415|Hsap
NP_002418|Hsap
NP_001451|Hsap
NP_001332890|Hsap
NP_001288276|Hsap
NP_001163873|Hsap
NP_001157908|Hsap
NP_001139206|Hsap
NP_001128619|Hsap
NP_001128618|Hsap
NP_000677|Hsap
NP_001004470|Hsap
XP_024100505|Hsap
XP_016866898|Hsap
XP_011536184|Hsap
XP_006715656|Hsap
XP_005268686|Hsap
NP_940980|Hsap
NP_940966|Hsap
NP_660350|Hsap
NP_009162|Hsap
NP_006260|Hsap
NP_004656|Hsap
NP_003847|Hsap
NP_003846|Hsap
NP_002967|Hsap
NP_001599|Hsap
NP_001358350|Hsap
NP_001352957|Hsap
NP_001269870|Hsap
NP_001258680|Hsap
NP_001229279|Hsap
NP_001007034|Hsap
NP_001128772|Hsap
NP_001191222|Hsap
NP_001019774|Hsap
NP_001191217|Hsap
XP_025257593|Hsap
NP_009122|Hsap
NP_001074006|Hsap
NP_001335685|Hsap
Cluster
Human proteins
Viral proteins
Species
12
10
8
6
4
2
Cluster
12
2
Human proteins
IFNG
AGTR2
ST8SIA6
CLEC6A
C7orf77
SNTN
TSPAN19
BCL2A1
SLC39A8
IL1RL1
CDC20B
TMEM212
MUC1
CLEC12A
VNN2
CALCRL
COL6A5
RTKN2
FMO2
SDR16C5
FAM216B
OVCH1
DNAH12
IL18R1
ROS1
CXCL10
ACADL
MMP13
SCN7A
RP1
WIF1
SLC6A14
DCSTAMP
ABCA13
DNAAF6
LRRK2
PIH1D3
Viral proteins
ORF1ab
ORF1a
spike protein S
ORF3a
ORF3b
ORF4a
ORF4b
ORF5
envelope protein E
membrane glycoprotein M
nucleocapsid protein N
ORF8b
Species
Hedgehogs
Humans

G.i

Overexpressed human genes of lung tissues that grouped with SARS-CoV-2, SARS and MERS of humans and animals. Significant differences of groups and counts of human genes are showed according to human and animal hosts in color or black. The differences observed were statistically tested using the chi-square test:

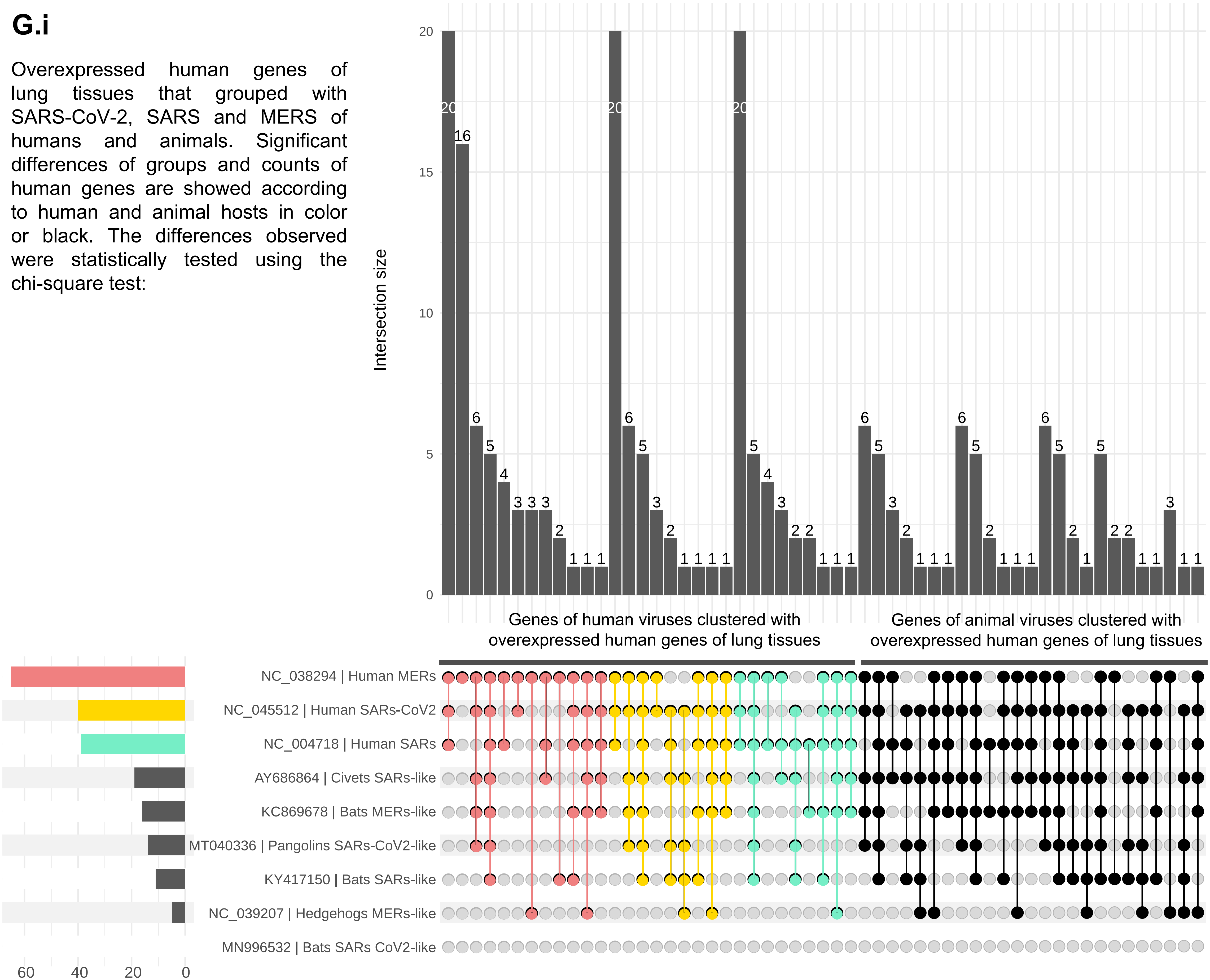

Human SARS-CoV-2 vs animal SARS-CoV-2-like (p-value = $1.039e^{-15}$). Human SARS vs animal SARS-like (p-value = $2.35e^{-12}$). Human MERS vs animal MERS-like (p-value = $6.5e^{-13}$). Differences among all the groups (p-value = $2.9e^{-09}$). Chi-square test - Kruskal-Wallis rank sum test and corrected FDR

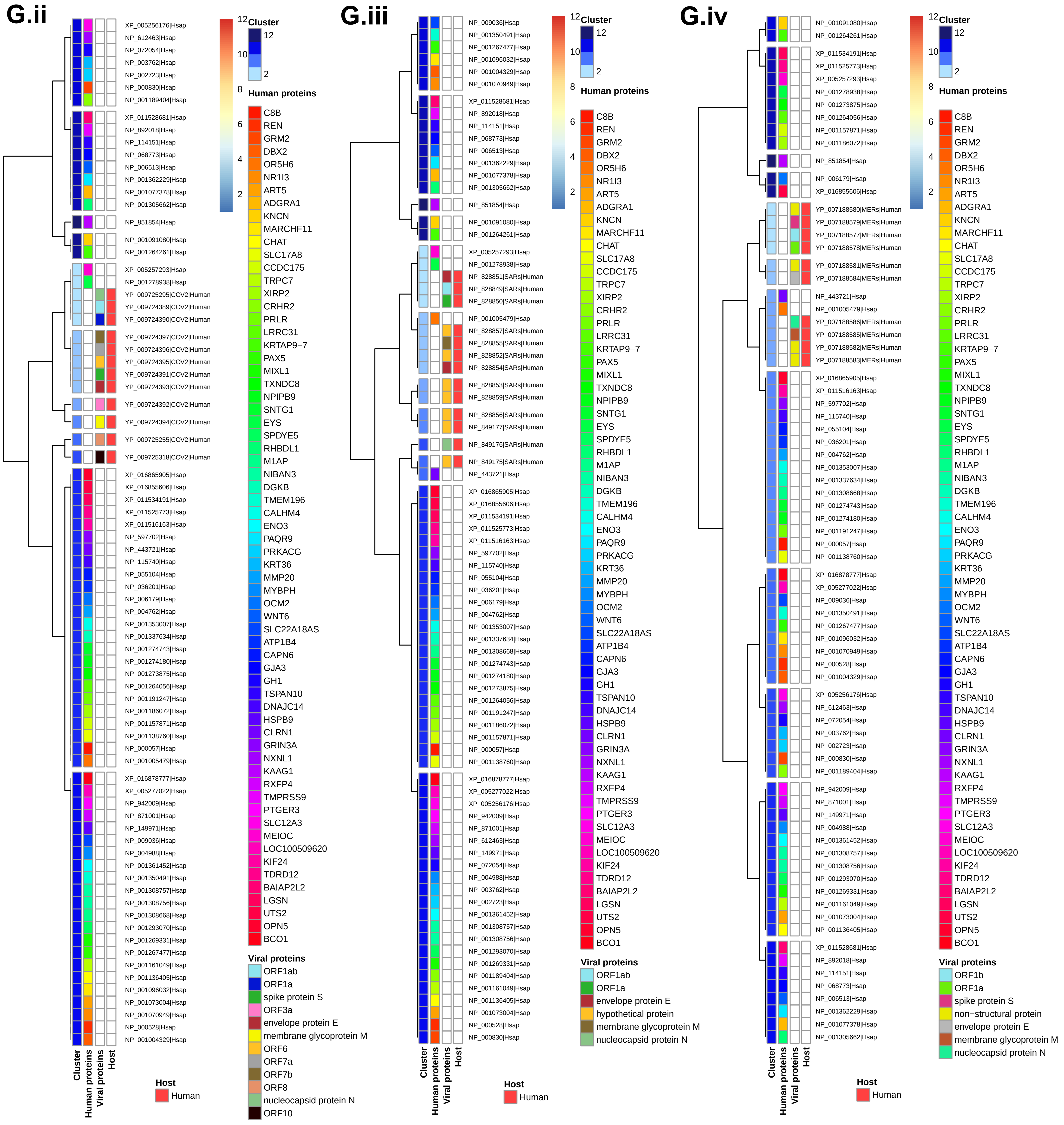

G.ii
XP_005256176|Hsap
NP_612463|Hsap
NP_072054|Hsap
NP_003762|Hsap
NP_002723|Hsap
NP_000830|Hsap
NP_001189404|Hsap
XP_011528681|Hsap
NP_892018|Hsap
NP_114151|Hsap
NP_068773|Hsap
NP_006513|Hsap
NP_001362229|Hsap
NP_001077378|Hsap
NP_001305662|Hsap
NP_851854|Hsap
NP_001091080|Hsap
NP_001264261|Hsap
XP_005257293|Hsap
NP_001278938|Hsap
YP_009725295|COV2|Human
YP_009724389|COV2|Human
YP_009724390|COV2|Human
YP_009724397|COV2|Human
YP_009724396|COV2|Human
YP_009724395|COV2|Human
YP_009724391|COV2|Human
YP_009724393|COV2|Human
YP_009724392|COV2|Human
YP_009724394|COV2|Human
YP_009725255|COV2|Human
YP_009725318|COV2|Human
XP_016865905|Hsap
XP_016855606|Hsap
XP_011534191|Hsap
XP_011525773|Hsap
XP_011516163|Hsap
NP_597702|Hsap
NP_443721|Hsap
NP_115740|Hsap
NP_055104|Hsap
NP_036201|Hsap
NP_006179|Hsap
NP_004762|Hsap
NP_001353007|Hsap
NP_001337634|Hsap
NP_001274743|Hsap
NP_001274180|Hsap
NP_001273875|Hsap
NP_001264056|Hsap
NP_001191247|Hsap
NP_001186072|Hsap
NP_001157871|Hsap
NP_001138760|Hsap
NP_000057|Hsap
NP_001005479|Hsap
XP_016878777|Hsap
XP_005277022|Hsap
NP_942009|Hsap
NP_871001|Hsap
NP_149971|Hsap
NP_009036|Hsap
NP_004988|Hsap
NP_001361452|Hsap
NP_001350491|Hsap
NP_001308757|Hsap
NP_001308756|Hsap
NP_001308668|Hsap
NP_001293070|Hsap
NP_001269331|Hsap
NP_001267477|Hsap
NP_001161049|Hsap
NP_001136405|Hsap
NP_001096032|Hsap
NP_001073004|Hsap
NP_001070949|Hsap
NP_000528|Hsap
NP_001004329|Hsap
Cluster
Human proteins
Viral proteins
Host
12
10
8
6
4
2
Cluster
12
2
Human proteins
C8B
REN
GRM2
DBX2
OR5H6
NR1I3
ART5
ADGRA1
KCNN
MARCHF11
CHAT
SLC17A8
CCDC175
TRPC7
XIRP2
CRHR2
PRLR
LRRC31
KRTAP9−7
PAX5
MIXL1
TXNDC8
NPIPB9
SNTG1
EYS
SPDYE5
RHBDL1
M1AP
NIBAN3
DGKB
TMEM196
CALHM4
ENO3
PAQR9
PRKACG
KRT36
MMP20
MYBPH
OCM2
WNT6
SLC22A18AS
ATP1B4
CAPN6
GJA3
GH1
TSPAN10
DNAJC14
HSPB9
CLRN1
GRIN3A
NXNL1
KAAG1
RXFP4
TMPRSS9
PTGER3
SLC12A3
MEIOC
LOC100509620
KIF24
TDRD12
BAIAP2L2
LGSN
UTS2
OPN5
BCO1
Viral proteins
ORF1ab
ORF1a
spike protein S
ORF3a
envelope protein E
membrane glycoprotein M
ORF6
ORF7a
ORF7b
ORF8
nucleocapsid protein N
ORF10
Host
Human
G.iii
NP_009036|Hsap
NP_001350491|Hsap
NP_001267477|Hsap
NP_001096032|Hsap
NP_001004329|Hsap
NP_001070949|Hsap
XP_011528681|Hsap
NP_892018|Hsap
NP_114151|Hsap
NP_068773|Hsap
NP_006513|Hsap
NP_001362229|Hsap
NP_001077378|Hsap
NP_001305662|Hsap
NP_851854|Hsap
NP_001091080|Hsap
NP_001264261|Hsap
XP_005257293|Hsap
NP_001278938|Hsap
NP_828851|SARs|Human
NP_828849|SARs|Human
NP_828850|SARs|Human
NP_001005479|Hsap
NP_828857|SARs|Human
NP_828855|SARs|Human
NP_828852|SARs|Human
NP_828854|SARs|Human
NP_828853|SARs|Human
NP_828859|SARs|Human
NP_828856|SARs|Human
NP_849177|SARs|Human
NP_849176|SARs|Human
NP_849175|SARs|Human
NP_443721|Hsap
XP_016865905|Hsap
XP_016855606|Hsap
XP_011534191|Hsap
XP_011525773|Hsap
XP_011516163|Hsap
NP_597702|Hsap
NP_115740|Hsap
NP_055104|Hsap
NP_036201|Hsap
NP_006179|Hsap
NP_004762|Hsap
NP_001353007|Hsap
NP_001337634|Hsap
NP_001308668|Hsap
NP_001274743|Hsap
NP_001274180|Hsap
NP_001273875|Hsap
NP_001264056|Hsap
NP_001191247|Hsap
NP_001186072|Hsap
NP_001157871|Hsap
NP_000057|Hsap
NP_001138760|Hsap
XP_016878777|Hsap
XP_005277022|Hsap
XP_005256176|Hsap
NP_942009|Hsap
NP_871001|Hsap
NP_612463|Hsap
NP_149971|Hsap
NP_072054|Hsap
NP_004988|Hsap
NP_003762|Hsap
NP_002723|Hsap
NP_001361452|Hsap
NP_001308757|Hsap
NP_001308756|Hsap
NP_001293070|Hsap
NP_001269331|Hsap
NP_001189404|Hsap
NP_001161049|Hsap
NP_001136405|Hsap
NP_001073004|Hsap
NP_000528|Hsap
NP_000830|Hsap
Cluster
Human proteins
Viral proteins
Host
12
10
8
6
4
2
Cluster
12
2
Human proteins
C8B
REN
GRM2
DBX2
OR5H6
NR1I3
ART5
ADGRA1
KCNN
MARCHF11
CHAT
SLC17A8
CCDC175
TRPC7
XIRP2
CRHR2
PRLR
LRRC31
KRTAP9−7
PAX5
MIXL1
TXNDC8
NPIPB9
SNTG1
EYS
SPDYE5
RHBDL1
M1AP
NIBAN3
DGKB
TMEM196
CALHM4
ENO3
PAQR9
PRKACG
KRT36
MMP20
MYBPH
OCM2
WNT6
SLC22A18AS
ATP1B4
CAPN6
GJA3
GH1
TSPAN10
DNAJC14
HSPB9
CLRN1
GRIN3A
NXNL1
KAAG1
RXFP4
TMPRSS9
PTGER3
SLC12A3
MEIOC
LOC100509620
KIF24
TDRD12
BAIAP2L2
LGSN
UTS2
OPN5
BCO1
Viral proteins
ORF1ab
ORF1a
envelope protein E
hypothetical protein
membrane glycoprotein M
nucleocapsid protein N
Host
Human
G.iv
NP_001091080|Hsap
NP_001264261|Hsap
XP_011534191|Hsap
XP_011525773|Hsap
XP_005257293|Hsap
NP_001278938|Hsap
NP_001273875|Hsap
NP_001264056|Hsap
NP_001157871|Hsap
NP_001186072|Hsap
NP_851854|Hsap
NP_006179|Hsap
XP_016855606|Hsap
YP_007188580|MERs|Human
YP_007188579|MERs|Human
YP_007188577|MERs|Human
YP_007188578|MERs|Human
YP_007188581|MERs|Human
YP_007188584|MERs|Human
NP_443721|Hsap
NP_001005479|Hsap
YP_007188586|MERs|Human
YP_007188585|MERs|Human
YP_007188582|MERs|Human
YP_007188583|MERs|Human
XP_016865905|Hsap
XP_011516163|Hsap
NP_597702|Hsap
NP_115740|Hsap
NP_055104|Hsap
NP_036201|Hsap
NP_004762|Hsap
NP_001353007|Hsap
NP_001337634|Hsap
NP_001308668|Hsap
NP_001274743|Hsap
NP_001274180|Hsap
NP_001191247|Hsap
NP_000057|Hsap
NP_001138760|Hsap
XP_016878777|Hsap
XP_005277022|Hsap
NP_009036|Hsap
NP_001350491|Hsap
NP_001267477|Hsap
NP_001096032|Hsap
NP_001070949|Hsap
NP_000528|Hsap
NP_001004329|Hsap
XP_005256176|Hsap
NP_612463|Hsap
NP_072054|Hsap
NP_003762|Hsap
NP_002723|Hsap
NP_000830|Hsap
NP_001189404|Hsap
NP_942009|Hsap
NP_871001|Hsap
NP_149971|Hsap
NP_004988|Hsap
NP_001361452|Hsap
NP_001308757|Hsap
NP_001308756|Hsap
NP_001293070|Hsap
NP_001269331|Hsap
NP_001161049|Hsap
NP_001073004|Hsap
NP_001136405|Hsap
XP_011528681|Hsap
NP_892018|Hsap
NP_114151|Hsap
NP_068773|Hsap
NP_006513|Hsap
NP_001362229|Hsap
NP_001077378|Hsap
NP_001305662|Hsap
Cluster
Human proteins
Viral proteins
Host
12
10
8
6
4
2
Cluster
12
2
Human proteins
C8B
REN
GRM2
DBX2
OR5H6
NR1I3
ART5
ADGRA1
KCNN
MARCHF11
CHAT
SLC17A8
CCDC175
TRPC7
XIRP2
CRHR2
PRLR
LRRC31
KRTAP9−7
PAX5
MIXL1
TXNDC8
NPIPB9
SNTG1
EYS
SPDYE5
RHBDL1
M1AP
NIBAN3
DGKB
TMEM196
CALHM4
ENO3
PAQR9
PRKACG
KRT36
MMP20
MYBPH
OCM2
WNT6
SLC22A18AS
ATP1B4
CAPN6
GJA3
GH1
TSPAN10
DNAJC14
HSPB9
CLRN1
GRIN3A
NXNL1
KAAG1
RXFP4
TMPRSS9
PTGER3
SLC12A3
MEIOC
LOC100509620
KIF24
TDRD12
BAIAP2L2
LGSN
UTS2
OPN5
BCO1
Viral proteins
ORF1b
ORF1a
spike protein S
non−structural protein
envelope protein E
membrane glycoprotein M
nucleocapsid protein N
Host
Human

H.i

Under-expressed human genes of lung tissues that grouped with SARS-CoV-2, SARS and MERS of humans and animals. Significant differences of groups and counts of human genes are showed according to human and animal hosts in color or black. The differences observed were statistically tested using the chi-square test.

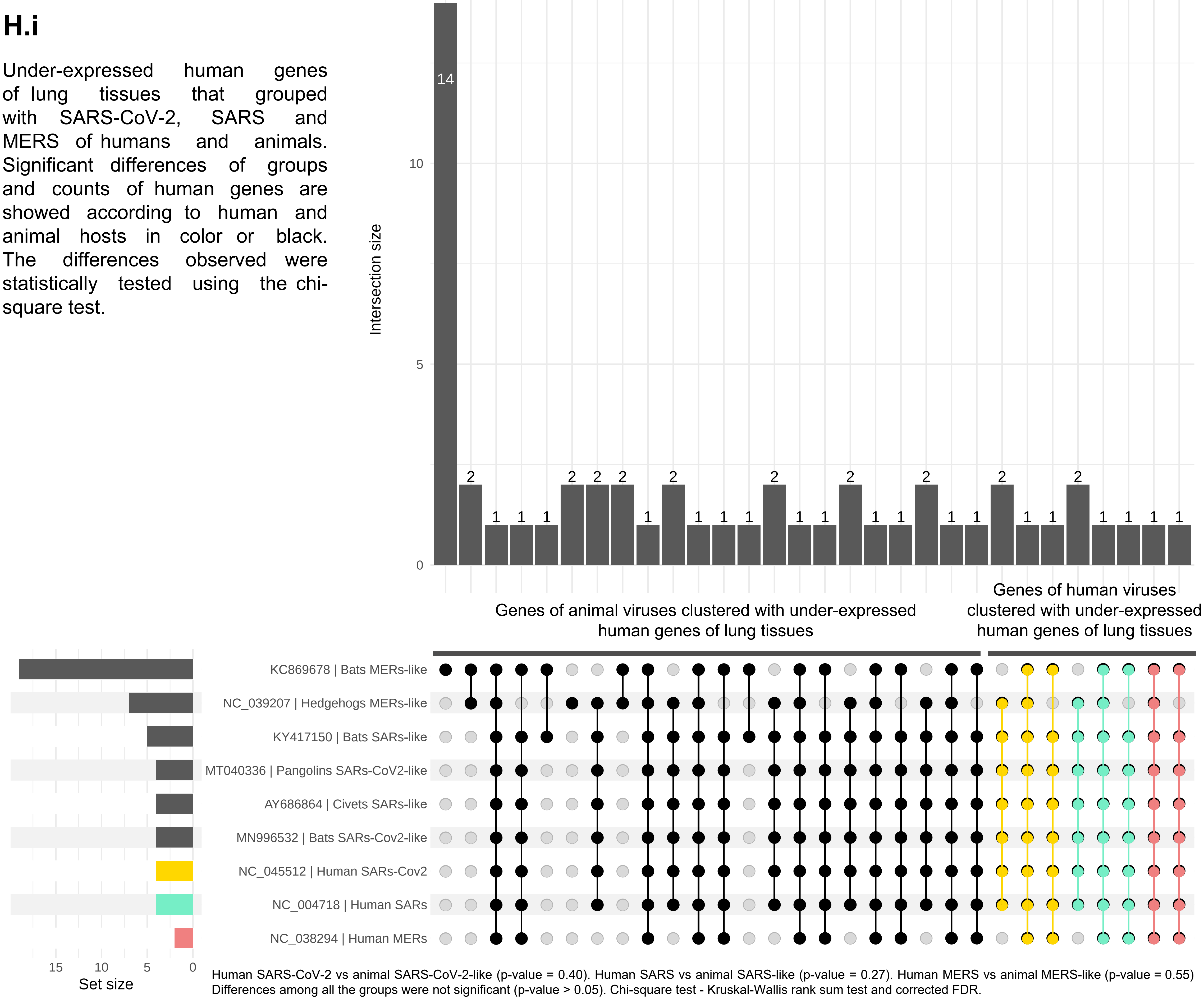

Human SARS-CoV-2 vs animal SARS-CoV-2-like (p-value = 0.40). Human SARS vs animal SARS-like (p-value = 0.27). Human MERS vs animal MERS-like (p-value = 0.55) Differences among all the groups were not significant (p-value > 0.05). Chi-square test - Kruskal-Wallis rank sum test and corrected FDR.

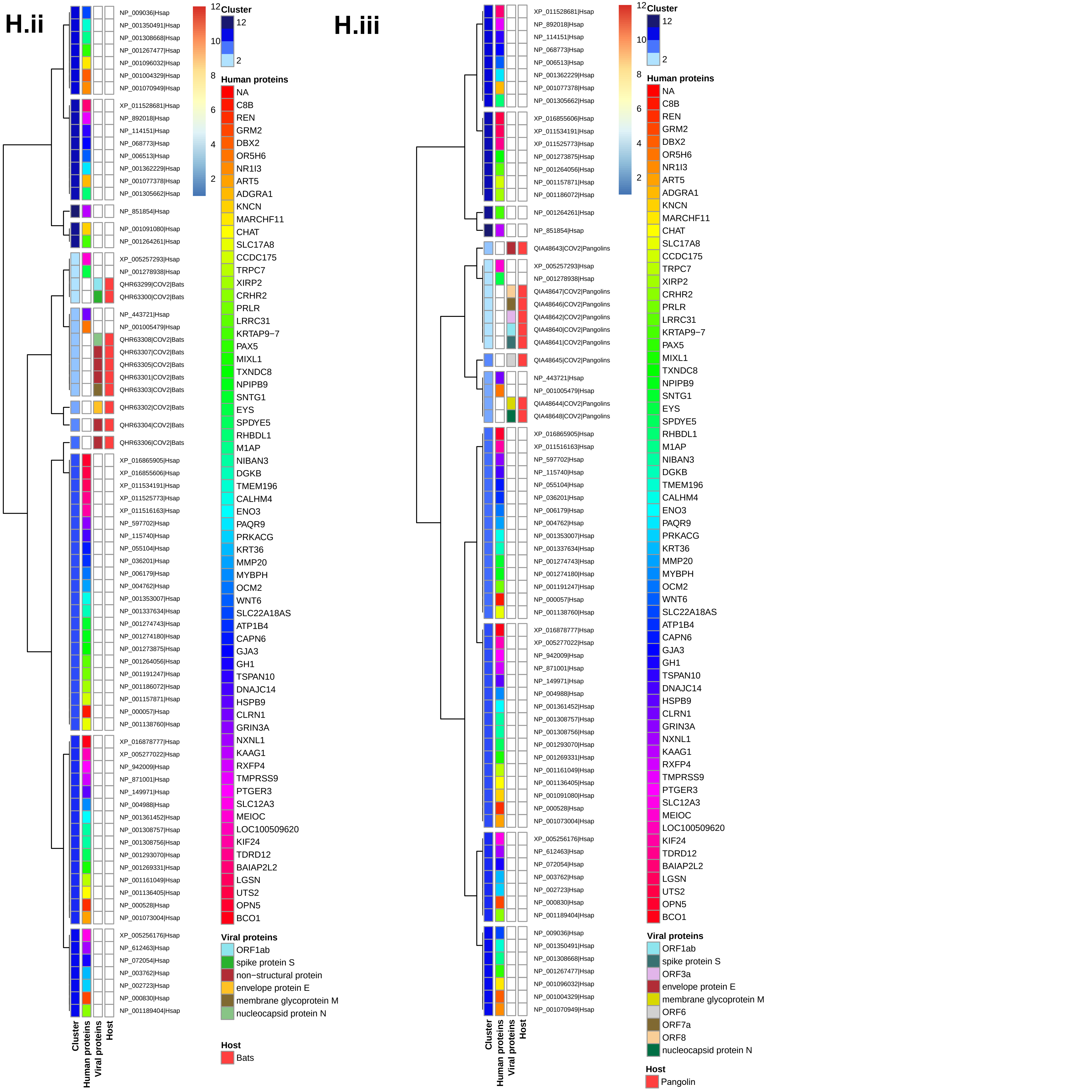
H.ii
NP_009036|Hsap
NP_001350491|Hsap
NP_001308668|Hsap
NP_001267477|Hsap
NP_001096032|Hsap
NP_001004329|Hsap
NP_001070949|Hsap
XP_011528681|Hsap
NP_892018|Hsap
NP_114151|Hsap
NP_068773|Hsap
NP_006513|Hsap
NP_001362229|Hsap
NP_001077378|Hsap
NP_001305662|Hsap
NP_851854|Hsap
NP_001091080|Hsap
NP_001264261|Hsap
XP_005257293|Hsap
NP_001278938|Hsap
QHR63299|COV2|Bats
QHR63300|COV2|Bats
NP_443721|Hsap
NP_001005479|Hsap
QHR63308|COV2|Bats
QHR63307|COV2|Bats
QHR63305|COV2|Bats
QHR63301|COV2|Bats
QHR63303|COV2|Bats
QHR63302|COV2|Bats
QHR63304|COV2|Bats
QHR63306|COV2|Bats
XP_016865905|Hsap
XP_016855606|Hsap
XP_011534191|Hsap
XP_011525773|Hsap
XP_011516163|Hsap
NP_597702|Hsap
NP_115740|Hsap
NP_055104|Hsap
NP_036201|Hsap
NP_006179|Hsap
NP_004762|Hsap
NP_001353007|Hsap
NP_001337634|Hsap
NP_001274743|Hsap
NP_001274180|Hsap
NP_001273875|Hsap
NP_001264056|Hsap
NP_001191247|Hsap
NP_001186072|Hsap
NP_001157871|Hsap
NP_000057|Hsap
NP_001138760|Hsap
XP_016878777|Hsap
XP_005277022|Hsap
NP_942009|Hsap
NP_871001|Hsap
NP_149971|Hsap
NP_004988|Hsap
NP_001361452|Hsap
NP_001308757|Hsap
NP_001308756|Hsap
NP_001293070|Hsap
NP_001269331|Hsap
NP_001161049|Hsap
NP_001136405|Hsap
NP_000528|Hsap
NP_001073004|Hsap
XP_005256176|Hsap
NP_612463|Hsap
NP_072054|Hsap
NP_003762|Hsap
NP_002723|Hsap
NP_000830|Hsap
NP_001189404|Hsap
Cluster
Human proteins
Viral proteins
Host
12
10
8
6
4
2
Cluster
12
2
Human proteins
NA
C8B
REN
GRM2
DBX2
OR5H6
NR1I3
ART5
ADGRA1
KNCN
MARCHF11
CHAT
SLC17A8
CCDC175
TRPC7
XIRP2
CRHR2
PRLR
LRRC31
KRTAP9−7
PAX5
MIXL1
TXNDC8
NPIPB9
SNTG1
EYS
SPDYE5
RHBDL1
M1AP
NIBAN3
DGKB
TMEM196
CALHM4
ENO3
PAQR9
PRKACG
KRT36
MMP20
MYBPH
OCM2
WNT6
SLC22A18AS
ATP1B4
CAPN6
GJA3
GH1
TSPAN10
DNAJC14
HSPB9
CLRN1
GRIN3A
NXNL1
KAAG1
RXFP4
TMPRSS9
PTGER3
SLC12A3
MEIOC
LOC100509620
KIF24
TDRD12
BAIAP2L2
LGSN
UTS2
OPN5
BCO1
Viral proteins
ORF1ab
spike protein S
non−structural protein
envelope protein E
membrane glycoprotein M
nucleocapsid protein N
Host
Bats
H.iii
XP_011528681|Hsap
NP_892018|Hsap
NP_114151|Hsap
NP_068773|Hsap
NP_006513|Hsap
NP_001362229|Hsap
NP_001077378|Hsap
NP_001305662|Hsap
XP_016855606|Hsap
XP_011534191|Hsap
XP_011525773|Hsap
NP_001273875|Hsap
NP_001264056|Hsap
NP_001157871|Hsap
NP_001186072|Hsap
NP_001264261|Hsap
NP_851854|Hsap
QIA48643|COV2|Pangolins
XP_005257293|Hsap
NP_001278938|Hsap
QIA48647|COV2|Pangolins
QIA48646|COV2|Pangolins
QIA48642|COV2|Pangolins
QIA48640|COV2|Pangolins
QIA48641|COV2|Pangolins
QIA48645|COV2|Pangolins
NP_443721|Hsap
NP_001005479|Hsap
QIA48644|COV2|Pangolins
QIA48648|COV2|Pangolins
XP_016865905|Hsap
XP_011516163|Hsap
NP_597702|Hsap
NP_115740|Hsap
NP_055104|Hsap
NP_036201|Hsap
NP_006179|Hsap
NP_004762|Hsap
NP_001353007|Hsap
NP_001337634|Hsap
NP_001274743|Hsap
NP_001274180|Hsap
NP_001191247|Hsap
NP_000057|Hsap
NP_001138760|Hsap
XP_016878777|Hsap
XP_005277022|Hsap
NP_942009|Hsap
NP_871001|Hsap
NP_149971|Hsap
NP_004988|Hsap
NP_001361452|Hsap
NP_001308757|Hsap
NP_001308756|Hsap
NP_001293070|Hsap
NP_001269331|Hsap
NP_001161049|Hsap
NP_001136405|Hsap
NP_001091080|Hsap
NP_000528|Hsap
NP_001073004|Hsap
XP_005256176|Hsap
NP_612463|Hsap
NP_072054|Hsap
NP_003762|Hsap
NP_002723|Hsap
NP_000830|Hsap
NP_001189404|Hsap
NP_009036|Hsap
NP_001350491|Hsap
NP_001308668|Hsap
NP_001267477|Hsap
NP_001096032|Hsap
NP_001004329|Hsap
NP_001070949|Hsap
Cluster
Human proteins
Viral proteins
Host
12
10
8
6
4
2
Cluster
12
2
Human proteins
NA
C8B
REN
GRM2
DBX2
OR5H6
NR1I3
ART5
ADGRA1
KNCN
MARCHF11
CHAT
SLC17A8
CCDC175
TRPC7
XIRP2
CRHR2
PRLR
LRRC31
KRTAP9−7
PAX5
MIXL1
TXNDC8
NPIPB9
SNTG1
EYS
SPDYE5
RHBDL1
M1AP
NIBAN3
DGKB
TMEM196
CALHM4
ENO3
PAQR9
PRKACG
KRT36
MMP20
MYBPH
OCM2
WNT6
SLC22A18AS
ATP1B4
CAPN6
GJA3
GH1
TSPAN10
DNAJC14
HSPB9
CLRN1
GRIN3A
NXNL1
KAAG1
RXFP4
TMPRSS9
PTGER3
SLC12A3
MEIOC
LOC100509620
KIF24
TDRD12
BAIAP2L2
LGSN
UTS2
OPN5
BCO1
Viral proteins
ORF1ab
spike protein S
ORF3a
envelope protein E
membrane glycoprotein M
ORF6
ORF7a
ORF8
nucleocapsid protein N
Host
Pangolin

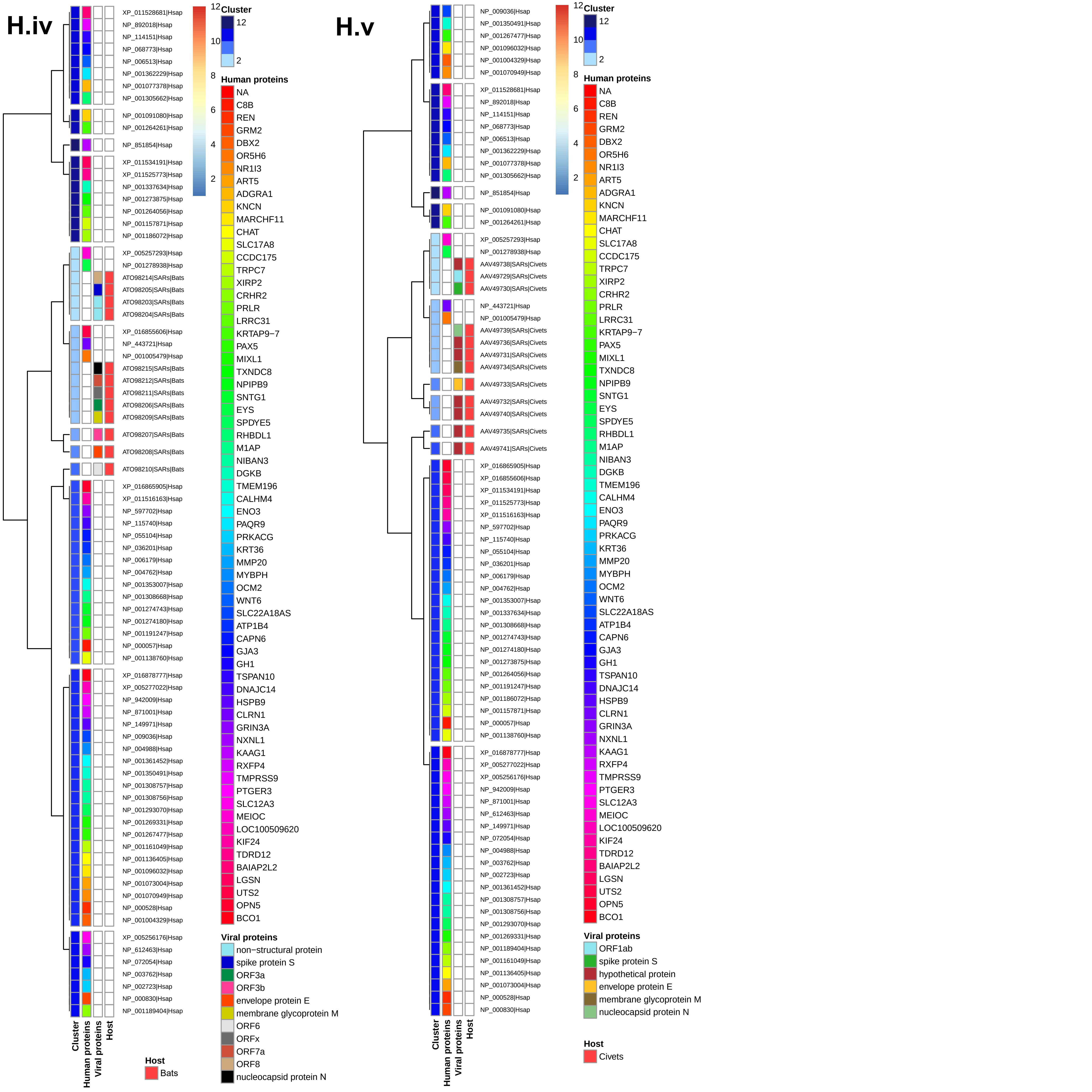

H.iv
XP_011528681|Hsap
NP_892018|Hsap
NP_114151|Hsap
NP_068773|Hsap
NP_006513|Hsap
NP_001362229|Hsap
NP_001077378|Hsap
NP_001305662|Hsap
NP_001091080|Hsap
NP_001264261|Hsap
NP_851854|Hsap
XP_011534191|Hsap
XP_011525773|Hsap
NP_001337634|Hsap
NP_001273875|Hsap
NP_001264056|Hsap
NP_001157871|Hsap
NP_001186072|Hsap
XP_005257293|Hsap
NP_001278938|Hsap
ATO98214|SARs|Bats
ATO98205|SARs|Bats
ATO98203|SARs|Bats
ATO98204|SARs|Bats
XP_016855606|Hsap
NP_443721|Hsap
NP_001005479|Hsap
ATO98215|SARs|Bats
ATO98212|SARs|Bats
ATO98211|SARs|Bats
ATO98206|SARs|Bats
ATO98209|SARs|Bats
ATO98207|SARs|Bats
ATO98208|SARs|Bats
ATO98210|SARs|Bats
XP_016865905|Hsap
XP_011516163|Hsap
NP_597702|Hsap
NP_115740|Hsap
NP_055104|Hsap
NP_036201|Hsap
NP_006179|Hsap
NP_004762|Hsap
NP_001353007|Hsap
NP_001308668|Hsap
NP_001274743|Hsap
NP_001274180|Hsap
NP_001191247|Hsap
NP_000057|Hsap
NP_001138760|Hsap
XP_016878777|Hsap
XP_005277022|Hsap
NP_942009|Hsap
NP_871001|Hsap
NP_149971|Hsap
NP_009036|Hsap
NP_004988|Hsap
NP_001361452|Hsap
NP_001350491|Hsap
NP_001308757|Hsap
NP_001308756|Hsap
NP_001293070|Hsap
NP_001269331|Hsap
NP_001267477|Hsap
NP_001161049|Hsap
NP_001136405|Hsap
NP_001096032|Hsap
NP_001073004|Hsap
NP_001070949|Hsap
NP_000528|Hsap
NP_001004329|Hsap
XP_005256176|Hsap
NP_612463|Hsap
NP_072054|Hsap
NP_003762|Hsap
NP_002723|Hsap
NP_000830|Hsap
NP_001189404|Hsap
Cluster
Human proteins
Viral proteins
Host
12
10
8
6
4
2
Cluster
12
2
Human proteins
NA
C8B
REN
GRM2
DBX2
OR5H6
NR1I3
ART5
ADGRA1
KNCN
MARCHF11
CHAT
SLC17A8
CCDC175
TRPC7
XIRP2
CRHR2
PRLR
LRRC31
KRTAP9−7
PAX5
MIXL1
TXNDC8
NPIPB9
SNTG1
EYS
SPDYE5
RHBDL1
M1AP
NIBAN3
DGKB
TMEM196
CALHM4
ENO3
PAQR9
PRKACG
KRT36
MMP20
MYBPH
OCM2
WNT6
SLC22A18AS
ATP1B4
CAPN6
GJA3
GH1
TSPAN10
DNAJC14
HSPB9
CLRN1
GRIN3A
NXNL1
KAAG1
RXFP4
TMPRSS9
PTGER3
SLC12A3
MEIOC
LOC100509620
KIF24
TDRD12
BAIAP2L2
LGSN
UTS2
OPN5
BCO1
Viral proteins
non−structural protein
spike protein S
ORF3a
ORF3b
envelope protein E
membrane glycoprotein M
ORF6
ORFx
ORF7a
ORF8
nucleocapsid protein N
Host
Bats
H.v
NP_009036|Hsap
NP_001350491|Hsap
NP_001267477|Hsap
NP_001096032|Hsap
NP_001004329|Hsap
NP_001070949|Hsap
XP_011528681|Hsap
NP_892018|Hsap
NP_114151|Hsap
NP_068773|Hsap
NP_006513|Hsap
NP_001362229|Hsap
NP_001077378|Hsap
NP_001305662|Hsap
NP_851854|Hsap
NP_001091080|Hsap
NP_001264261|Hsap
XP_005257293|Hsap
NP_001278938|Hsap
AAV49738|SARs|Civets
AAV49729|SARs|Civets
AAV49730|SARs|Civets
NP_443721|Hsap
NP_001005479|Hsap
AAV49739|SARs|Civets
AAV49736|SARs|Civets
AAV49731|SARs|Civets
AAV49734|SARs|Civets
AAV49733|SARs|Civets
AAV49732|SARs|Civets
AAV49740|SARs|Civets
AAV49735|SARs|Civets
AAV49741|SARs|Civets
XP_016865905|Hsap
XP_016855606|Hsap
XP_011534191|Hsap
XP_011525773|Hsap
XP_011516163|Hsap
NP_597702|Hsap
NP_115740|Hsap
NP_055104|Hsap
NP_036201|Hsap
NP_006179|Hsap
NP_004762|Hsap
NP_001353007|Hsap
NP_001337634|Hsap
NP_001308668|Hsap
NP_001274743|Hsap
NP_001274180|Hsap
NP_001273875|Hsap
NP_001264056|Hsap
NP_001191247|Hsap
NP_001186072|Hsap
NP_001157871|Hsap
NP_000057|Hsap
NP_001138760|Hsap
XP_016878777|Hsap
XP_005277022|Hsap
XP_005256176|Hsap
NP_942009|Hsap
NP_871001|Hsap
NP_612463|Hsap
NP_149971|Hsap
NP_072054|Hsap
NP_004988|Hsap
NP_003762|Hsap
NP_002723|Hsap
NP_001361452|Hsap
NP_001308757|Hsap
NP_001308756|Hsap
NP_001293070|Hsap
NP_001269331|Hsap
NP_001189404|Hsap
NP_001161049|Hsap
NP_001136405|Hsap
NP_001073004|Hsap
NP_000528|Hsap
NP_000830|Hsap
Cluster
Human proteins
Viral proteins
Host
12
10
8
6
4
2
Cluster
12
2
Human proteins
NA
C8B
REN
GRM2
DBX2
OR5H6
NR1I3
ART5
ADGRA1
KNCN
MARCHF11
CHAT
SLC17A8
CCDC175
TRPC7
XIRP2
CRHR2
PRLR
LRRC31
KRTAP9−7
PAX5
MIXL1
TXNDC8
NPIPB9
SNTG1
EYS
SPDYE5
RHBDL1
M1AP
NIBAN3
DGKB
TMEM196
CALHM4
ENO3
PAQR9
PRKACG
KRT36
MMP20
MYBPH
OCM2
WNT6
SLC22A18AS
ATP1B4
CAPN6
GJA3
GH1
TSPAN10
DNAJC14
HSPB9
CLRN1
GRIN3A
NXNL1
KAAG1
RXFP4
TMPRSS9
PTGER3
SLC12A3
MEIOC
LOC100509620
KIF24
TDRD12
BAIAP2L2
LGSN
UTS2
OPN5
BCO1
Viral proteins
ORF1ab
spike protein S
hypothetical protein
envelope protein E
membrane glycoprotein M
nucleocapsid protein N
Host
Civets

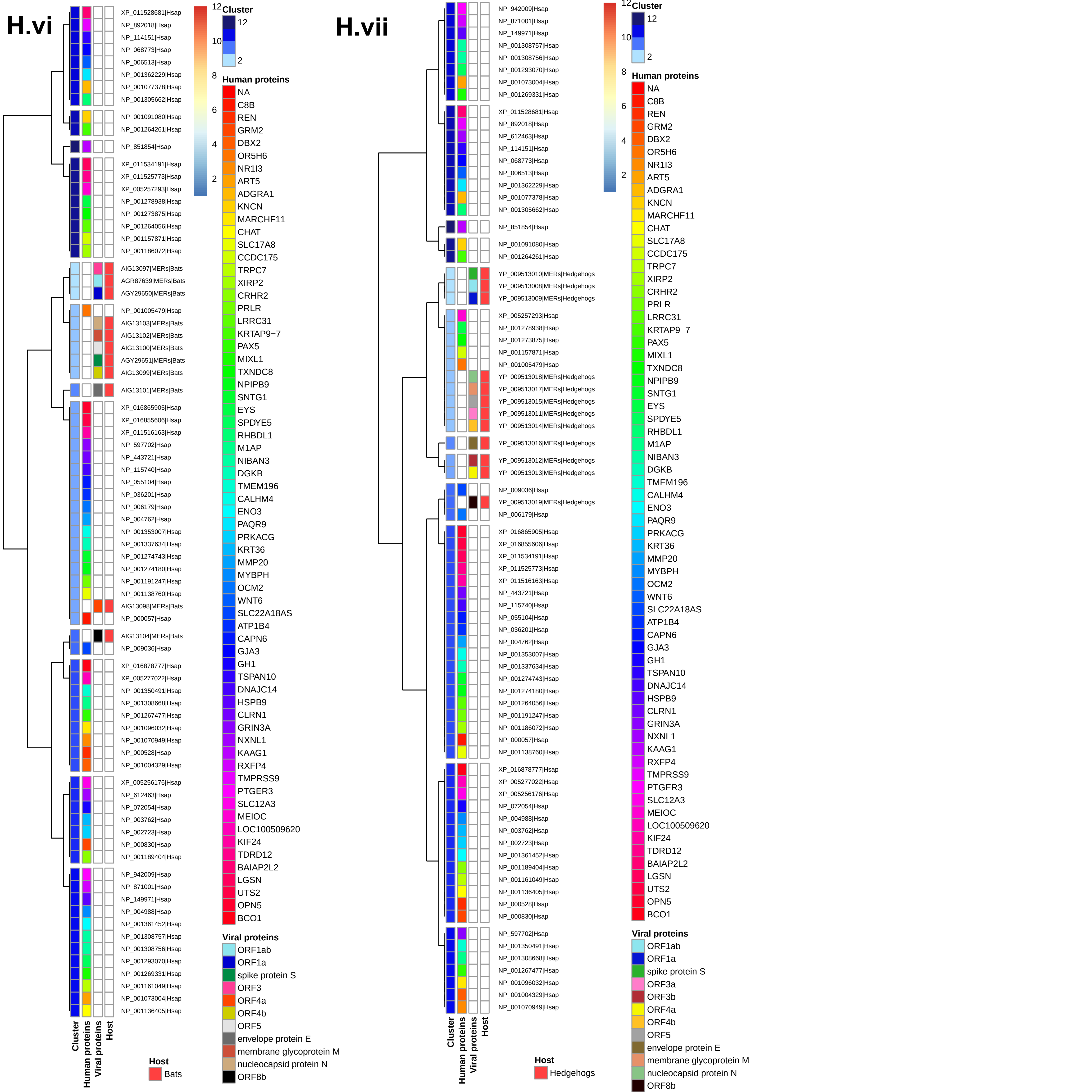
H.vi
XP_011528681|Hsap
NP_892018|Hsap
NP_114151|Hsap
NP_068773|Hsap
NP_006513|Hsap
NP_001362229|Hsap
NP_001077378|Hsap
NP_001305662|Hsap
NP_001091080|Hsap
NP_001264261|Hsap
NP_851854|Hsap
XP_011534191|Hsap
XP_011525773|Hsap
XP_005257293|Hsap
NP_001278938|Hsap
NP_001273875|Hsap
NP_001264056|Hsap
NP_001157871|Hsap
NP_001186072|Hsap
AIG13097|MERs|Bats
AGR87639|MERs|Bats
AGY29650|MERs|Bats
NP_001005479|Hsap
AIG13103|MERs|Bats
AIG13102|MERs|Bats
AIG13100|MERs|Bats
AGY29651|MERs|Bats
AIG13099|MERs|Bats
AIG13101|MERs|Bats
XP_016865905|Hsap
XP_016855606|Hsap
XP_011516163|Hsap
NP_597702|Hsap
NP_443721|Hsap
NP_115740|Hsap
NP_055104|Hsap
NP_036201|Hsap
NP_006179|Hsap
NP_004762|Hsap
NP_001353007|Hsap
NP_001337634|Hsap
NP_001274743|Hsap
NP_001274180|Hsap
NP_001191247|Hsap
NP_001138760|Hsap
AIG13098|MERs|Bats
NP_000057|Hsap
AIG13104|MERs|Bats
NP_009036|Hsap
XP_016878777|Hsap
XP_005277022|Hsap
NP_001350491|Hsap
NP_001308668|Hsap
NP_001267477|Hsap
NP_001096032|Hsap
NP_001070949|Hsap
NP_000528|Hsap
NP_001004329|Hsap
XP_005256176|Hsap
NP_612463|Hsap
NP_072054|Hsap
NP_003762|Hsap
NP_002723|Hsap
NP_000830|Hsap
NP_001189404|Hsap
NP_942009|Hsap
NP_871001|Hsap
NP_149971|Hsap
NP_004988|Hsap
NP_001361452|Hsap
NP_001308757|Hsap
NP_001308756|Hsap
NP_001293070|Hsap
NP_001269331|Hsap
NP_001161049|Hsap
NP_001073004|Hsap
NP_001136405|Hsap
Cluster
Human proteins
Viral proteins
Host
12
10
8
6
4
2
Cluster
12
2
Human proteins
NA
C8B
REN
GRM2
DBX2
OR5H6
NR1I3
ART5
ADGRA1
KNCN
MARCHF11
CHAT
SLC17A8
CCDC175
TRPC7
XIRP2
CRHR2
PRLR
LRRC31
KRTAP9−7
PAX5
MIXL1
TXNDC8
NPIPB9
SNTG1
EYS
SPDYE5
RHBDL1
M1AP
NIBAN3
DGKB
TMEM196
CALHM4
ENO3
PAQR9
PRKACG
KRT36
MMP20
MYBPH
OCM2
WNT6
SLC22A18AS
ATP1B4
CAPN6
GJA3
GH1
TSPAN10
DNAJC14
HSPB9
CLRN1
GRIN3A
NXNL1
KAAG1
RXFP4
TMPRSS9
PTGER3
SLC12A3
MEIOC
LOC100509620
KIF24
TDRD12
BAIAP2L2
LGSN
UTS2
OPN5
BCO1
Viral proteins
ORF1ab
ORF1a
spike protein S
ORF3
ORF4a
ORF4b
ORF5
envelope protein E
membrane glycoprotein M
nucleocapsid protein N
ORF8b
Host
Bats
H.vii
NP_942009|Hsap
NP_871001|Hsap
NP_149971|Hsap
NP_001308757|Hsap
NP_001308756|Hsap
NP_001293070|Hsap
NP_001073004|Hsap
NP_001269331|Hsap
XP_011528681|Hsap
NP_892018|Hsap
NP_612463|Hsap
NP_114151|Hsap
NP_068773|Hsap
NP_006513|Hsap
NP_001362229|Hsap
NP_001077378|Hsap
NP_001305662|Hsap
NP_851854|Hsap
NP_001091080|Hsap
NP_001264261|Hsap
YP_009513010|MERs|Hedgehogs
YP_009513008|MERs|Hedgehogs
YP_009513009|MERs|Hedgehogs
XP_005257293|Hsap
NP_001278938|Hsap
NP_001273875|Hsap
NP_001157871|Hsap
NP_001005479|Hsap
YP_009513018|MERs|Hedgehogs
YP_009513017|MERs|Hedgehogs
YP_009513015|MERs|Hedgehogs
YP_009513011|MERs|Hedgehogs
YP_009513014|MERs|Hedgehogs
YP_009513016|MERs|Hedgehogs
YP_009513012|MERs|Hedgehogs
YP_009513013|MERs|Hedgehogs
NP_009036|Hsap
YP_009513019|MERs|Hedgehogs
NP_006179|Hsap
XP_016865905|Hsap
XP_016855606|Hsap
XP_011534191|Hsap
XP_011525773|Hsap
XP_011516163|Hsap
NP_443721|Hsap
NP_115740|Hsap
NP_055104|Hsap
NP_036201|Hsap
NP_004762|Hsap
NP_001353007|Hsap
NP_001337634|Hsap
NP_001274743|Hsap
NP_001274180|Hsap
NP_001264056|Hsap
NP_001191247|Hsap
NP_001186072|Hsap
NP_000057|Hsap
NP_001138760|Hsap
XP_016878777|Hsap
XP_005277022|Hsap
XP_005256176|Hsap
NP_072054|Hsap
NP_004988|Hsap
NP_003762|Hsap
NP_002723|Hsap
NP_001361452|Hsap
NP_001189404|Hsap
NP_001161049|Hsap
NP_001136405|Hsap
NP_000528|Hsap
NP_000830|Hsap
NP_597702|Hsap
NP_001350491|Hsap
NP_001308668|Hsap
NP_001267477|Hsap
NP_001096032|Hsap
NP_001004329|Hsap
NP_001070949|Hsap
Cluster
Human proteins
Viral proteins
Host
12
10
8
6
4
2
Cluster
12
2
Human proteins
NA
C8B
REN
GRM2
DBX2
OR5H6
NR1I3
ART5
ADGRA1
KNCN
MARCHF11
CHAT
SLC17A8
CCDC175
TRPC7
XIRP2
CRHR2
PRLR
LRRC31
KRTAP9−7
PAX5
MIXL1
TXNDC8
NPIPB9
SNTG1
EYS
SPDYE5
RHBDL1
M1AP
NIBAN3
DGKB
TMEM196
CALHM4
ENO3
PAQR9
PRKACG
KRT36
MMP20
MYBPH
OCM2
WNT6
SLC22A18AS
ATP1B4
CAPN6
GJA3
GH1
TSPAN10
DNAJC14
HSPB9
CLRN1
GRIN3A
NXNL1
KAAG1
RXFP4
TMPRSS9
PTGER3
SLC12A3
MEIOC
LOC100509620
KIF24
TDRD12
BAIAP2L2
LGSN
UTS2
OPN5
BCO1
Viral proteins
ORF1ab
ORF1a
spike protein S
ORF3a
ORF3b
ORF4a
ORF4b
ORF5
envelope protein E
membrane glycoprotein M
nucleocapsid protein N
ORF8b
Host
Hedgehogs
