# Supplementary material for: Molecular features similarities between SARS-CoV-2, SARS, MERS and key human genes could favour the viral infections and trigger collateral effects
Source: Sci Rep. 2021 Feb 18;11:4108. doi: 10.1038/s41598-021-83595-1 (PMC7893037; doi:10.1038/s41598-021-83595-1)
Supplement: Supplementary file 6 — Supplementary Information 6. [file 41598_2021_83595_MOESM6_ESM.pdf]

Molecular Features Similarities Between SARS-CoV-2, SARS, MERS and Key Human Genes Could Favour The Viral Infections and Trigger Collateral Effects

Authors: Lucas L. Maldonado*; Andrea Mendoza Bertelli and Laura Kamenetzky

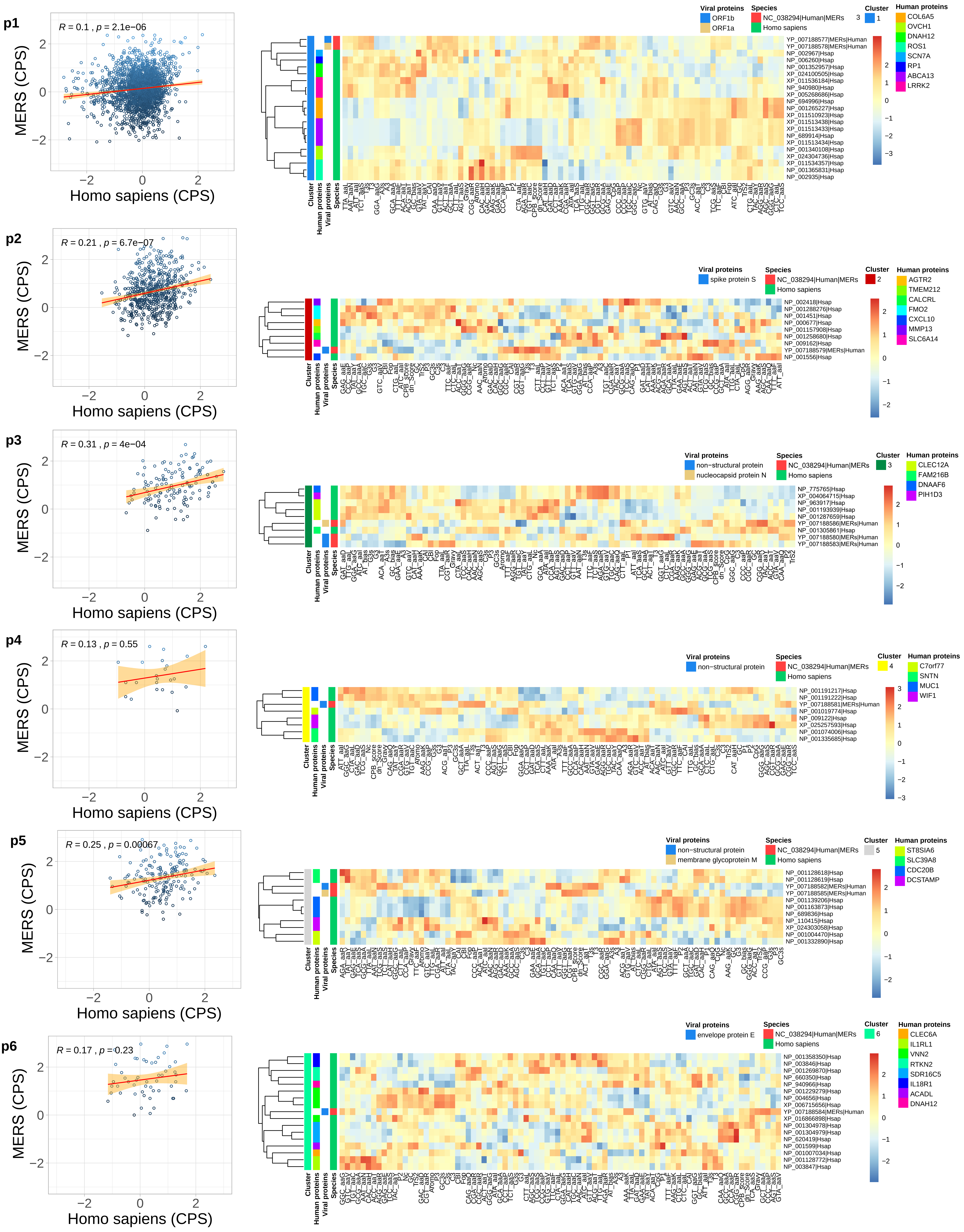

Supplementary file 6: Heatmap of clusters (1 to 6) using hierarchical method of viral genes for MERS (NC_038294) of human host and human genes based on the molecular features. CPB correlation is included in the left for each cluster relating the CPB of human genes (horizontal axis) and CPB of the viral genes (vertical axis).
